# Search for squarks and gluinos in final states with jets and missing transverse momentum at $\sqrt{s}$ =13 ${\mathrm{TeV}}$with the ATLAS detector

**DOI:** 10.1140/epjc/s10052-016-4184-8

**Published:** 2016-07-12

**Authors:** Atlas Collaboration, M. Aaboud, G. Aad, B. Abbott, J. Abdallah, O. Abdinov, B. Abeloos, R. Aben, O. S. AbouZeid, N. L. Abraham, H. Abramowicz, H. Abreu, R. Abreu, Y. Abulaiti, B. S. Acharya, S. Adachi, L. Adamczyk, D. L. Adams, J. Adelman, S. Adomeit, T. Adye, A. A. Affolder, T. Agatonovic-Jovin, J. Agricola, J. A. Aguilar-Saavedra, S. P. Ahlen, F. Ahmadov, G. Aielli, H. Akerstedt, T. P. A. Åkesson, A. V. Akimov, G. L. Alberghi, J. Albert, S. Albrand, M. J. Alconada Verzini, M. Aleksa, I. N. Aleksandrov, C. Alexa, G. Alexander, T. Alexopoulos, M. Alhroob, M. Aliev, G. Alimonti, J. Alison, S. P. Alkire, B. M. M. Allbrooke, B. W. Allen, P. P. Allport, A. Aloisio, A. Alonso, F. Alonso, C. Alpigiani, M. Alstaty, B. Alvarez Gonzalez, D. Álvarez Piqueras, M. G. Alviggi, B. T. Amadio, K. Amako, Y. Amaral Coutinho, C. Amelung, D. Amidei, S. P. Amor Dos Santos, A. Amorim, S. Amoroso, G. Amundsen, C. Anastopoulos, L. S. Ancu, N. Andari, T. Andeen, C. F. Anders, G. Anders, J. K. Anders, K. J. Anderson, A. Andreazza, V. Andrei, S. Angelidakis, I. Angelozzi, P. Anger, A. Angerami, F. Anghinolfi, A. V. Anisenkov, N. Anjos, A. Annovi, M. Antonelli, A. Antonov, F. Anulli, M. Aoki, L. Aperio Bella, G. Arabidze, Y. Arai, J. P. Araque, A. T. H. Arce, F. A. Arduh, J-F. Arguin, S. Argyropoulos, M. Arik, A. J. Armbruster, L. J. Armitage, O. Arnaez, H. Arnold, M. Arratia, O. Arslan, A. Artamonov, G. Artoni, S. Artz, S. Asai, N. Asbah, A. Ashkenazi, B. Åsman, L. Asquith, K. Assamagan, R. Astalos, M. Atkinson, N. B. Atlay, K. Augsten, G. Avolio, B. Axen, M. K. Ayoub, G. Azuelos, M. A. Baak, A. E. Baas, M. J. Baca, H. Bachacou, K. Bachas, M. Backes, M. Backhaus, P. Bagiacchi, P. Bagnaia, Y. Bai, J. T. Baines, O. K. Baker, E. M. Baldin, P. Balek, T. Balestri, F. Balli, W. K. Balunas, E. Banas, Sw. Banerjee, A. A. E. Bannoura, L. Barak, E. L. Barberio, D. Barberis, M. Barbero, T. Barillari, T. Barklow, N. Barlow, S. L. Barnes, B. M. Barnett, R. M. Barnett, Z. Barnovska, A. Baroncelli, G. Barone, A. J. Barr, L. Barranco Navarro, F. Barreiro, J. Barreiro Guimarães da Costa, R. Bartoldus, A. E. Barton, P. Bartos, A. Basalaev, A. Bassalat, R. L. Bates, S. J. Batista, J. R. Batley, M. Battaglia, M. Bauce, F. Bauer, H. S. Bawa, J. B. Beacham, M. D. Beattie, T. Beau, P. H. Beauchemin, P. Bechtle, H. P. Beck, K. Becker, M. Becker, M. Beckingham, C. Becot, A. J. Beddall, A. Beddall, V. A. Bednyakov, M. Bedognetti, C. P. Bee, L. J. Beemster, T. A. Beermann, M. Begel, J. K. Behr, C. Belanger-Champagne, A. S. Bell, G. Bella, L. Bellagamba, A. Bellerive, M. Bellomo, K. Belotskiy, O. Beltramello, N. L. Belyaev, O. Benary, D. Benchekroun, M. Bender, K. Bendtz, N. Benekos, Y. Benhammou, E. Benhar Noccioli, J. Benitez, D. P. Benjamin, J. R. Bensinger, S. Bentvelsen, L. Beresford, M. Beretta, D. Berge, E. Bergeaas Kuutmann, N. Berger, J. Beringer, S. Berlendis, N. R. Bernard, C. Bernius, F. U. Bernlochner, T. Berry, P. Berta, C. Bertella, G. Bertoli, F. Bertolucci, I. A. Bertram, C. Bertsche, D. Bertsche, G. J. Besjes, O. Bessidskaia Bylund, M. Bessner, N. Besson, C. Betancourt, S. Bethke, A. J. Bevan, W. Bhimji, R. M. Bianchi, L. Bianchini, M. Bianco, O. Biebel, D. Biedermann, R. Bielski, N. V. Biesuz, M. Biglietti, J. Bilbao De Mendizabal, H. Bilokon, M. Bindi, S. Binet, A. Bingul, C. Bini, S. Biondi, D. M. Bjergaard, C. W. Black, J. E. Black, K. M. Black, D. Blackburn, R. E. Blair, J.-B. Blanchard, J. E. Blanco, T. Blazek, I. Bloch, C. Blocker, W. Blum, U. Blumenschein, S. Blunier, G. J. Bobbink, V. S. Bobrovnikov, S. S. Bocchetta, A. Bocci, C. Bock, M. Boehler, D. Boerner, J. A. Bogaerts, D. Bogavac, A. G. Bogdanchikov, C. Bohm, V. Boisvert, P. Bokan, T. Bold, A. S. Boldyrev, M. Bomben, M. Bona, M. Boonekamp, A. Borisov, G. Borissov, J. Bortfeldt, D. Bortoletto, V. Bortolotto, K. Bos, D. Boscherini, M. Bosman, J. D. Bossio Sola, J. Boudreau, J. Bouffard, E. V. Bouhova-Thacker, D. Boumediene, C. Bourdarios, S. K. Boutle, A. Boveia, J. Boyd, I. R. Boyko, J. Bracinik, A. Brandt, G. Brandt, O. Brandt, U. Bratzler, B. Brau, J. E. Brau, H. M. Braun, W. D. Breaden Madden, K. Brendlinger, A. J. Brennan, L. Brenner, R. Brenner, S. Bressler, T. M. Bristow, D. Britton, D. Britzger, F. M. Brochu, I. Brock, R. Brock, G. Brooijmans, T. Brooks, W. K. Brooks, J. Brosamer, E. Brost, J. H Broughton, P. A. Bruckman de Renstrom, D. Bruncko, R. Bruneliere, A. Bruni, G. Bruni, B. H. Brunt, M. Bruschi, N. Bruscino, P. Bryant, L. Bryngemark, T. Buanes, Q. Buat, P. Buchholz, A. G. Buckley, I. A. Budagov, F. Buehrer, M. K. Bugge, O. Bulekov, D. Bullock, H. Burckhart, S. Burdin, C. D. Burgard, B. Burghgrave, K. Burka, S. Burke, I. Burmeister, E. Busato, D. Büscher, V. Büscher, P. Bussey, J. M. Butler, C. M. Buttar, J. M. Butterworth, P. Butti, W. Buttinger, A. Buzatu, A. R. Buzykaev, S. Cabrera Urbán, D. Caforio, V. M. Cairo, O. Cakir, N. Calace, P. Calafiura, A. Calandri, G. Calderini, P. Calfayan, L. P. Caloba, D. Calvet, S. Calvet, T. P. Calvet, R. Camacho Toro, S. Camarda, P. Camarri, D. Cameron, R. Caminal Armadans, C. Camincher, S. Campana, M. Campanelli, A. Camplani, A. Campoverde, V. Canale, A. Canepa, M. Cano Bret, J. Cantero, R. Cantrill, T. Cao, M. D. M. Capeans Garrido, I. Caprini, M. Caprini, M. Capua, R. Caputo, R. M. Carbone, R. Cardarelli, F. Cardillo, I. Carli, T. Carli, G. Carlino, L. Carminati, S. Caron, E. Carquin, G. D. Carrillo-Montoya, J. R. Carter, J. Carvalho, D. Casadei, M. P. Casado, M. Casolino, D. W. Casper, E. Castaneda-Miranda, R. Castelijn, A. Castelli, V. Castillo Gimenez, N. F. Castro, A. Catinaccio, J. R. Catmore, A. Cattai, J. Caudron, V. Cavaliere, E. Cavallaro, D. Cavalli, M. Cavalli-Sforza, V. Cavasinni, F. Ceradini, L. Cerda Alberich, B. C. Cerio, A. S. Cerqueira, A. Cerri, L. Cerrito, F. Cerutti, M. Cerv, A. Cervelli, S. A. Cetin, A. Chafaq, D. Chakraborty, S. K. Chan, Y. L. Chan, P. Chang, J. D. Chapman, D. G. Charlton, A. Chatterjee, C. C. Chau, C. A. Chavez Barajas, S. Che, S. Cheatham, A. Chegwidden, S. Chekanov, S. V. Chekulaev, G. A. Chelkov, M. A. Chelstowska, C. Chen, H. Chen, K. Chen, S. Chen, S. Chen, X. Chen, Y. Chen, H. C. Cheng, H. J Cheng, Y. Cheng, A. Cheplakov, E. Cheremushkina, R. Cherkaoui El Moursli, V. Chernyatin, E. Cheu, L. Chevalier, V. Chiarella, G. Chiarelli, G. Chiodini, A. S. Chisholm, A. Chitan, M. V. Chizhov, K. Choi, A. R. Chomont, S. Chouridou, B. K. B. Chow, V. Christodoulou, D. Chromek-Burckhart, J. Chudoba, A. J. Chuinard, J. J. Chwastowski, L. Chytka, G. Ciapetti, A. K. Ciftci, D. Cinca, V. Cindro, I. A. Cioara, A. Ciocio, F. Cirotto, Z. H. Citron, M. Citterio, M. Ciubancan, A. Clark, B. L. Clark, M. R. Clark, P. J. Clark, R. N. Clarke, C. Clement, Y. Coadou, M. Cobal, A. Coccaro, J. Cochran, L. Coffey, L. Colasurdo, B. Cole, A. P. Colijn, J. Collot, T. Colombo, G. Compostella, P. Conde Muiño, E. Coniavitis, S. H. Connell, I. A. Connelly, V. Consorti, S. Constantinescu, G. Conti, F. Conventi, M. Cooke, B. D. Cooper, A. M. Cooper-Sarkar, K. J. R. Cormier, T. Cornelissen, M. Corradi, F. Corriveau, A. Corso-Radu, A. Cortes-Gonzalez, G. Cortiana, G. Costa, M. J. Costa, D. Costanzo, G. Cottin, G. Cowan, B. E. Cox, K. Cranmer, S. J. Crawley, G. Cree, S. Crépé-Renaudin, F. Crescioli, W. A. Cribbs, M. Crispin Ortuzar, M. Cristinziani, V. Croft, G. Crosetti, T. Cuhadar Donszelmann, J. Cummings, M. Curatolo, J. Cúth, C. Cuthbert, H. Czirr, P. Czodrowski, G. D’amen, S. D’Auria, M. D’Onofrio, M. J. Da Cunha Sargedas De Sousa, C. Da Via, W. Dabrowski, T. Dado, T. Dai, O. Dale, F. Dallaire, C. Dallapiccola, M. Dam, J. R. Dandoy, N. P. Dang, A. C. Daniells, N. S. Dann, M. Danninger, M. Dano Hoffmann, V. Dao, G. Darbo, S. Darmora, J. Dassoulas, A. Dattagupta, W. Davey, C. David, T. Davidek, M. Davies, P. Davison, E. Dawe, I. Dawson, R. K. Daya-Ishmukhametova, K. De, R. de Asmundis, A. De Benedetti, S. De Castro, S. De Cecco, N. De Groot, P. de Jong, H. De la Torre, F. De Lorenzi, A. De Maria, D. De Pedis, A. De Salvo, U. De Sanctis, A. De Santo, J. B. De Vivie De Regie, W. J. Dearnaley, R. Debbe, C. Debenedetti, D. V. Dedovich, N. Dehghanian, I. Deigaard, M. Del Gaudio, J. Del Peso, T. Del Prete, D. Delgove, F. Deliot, C. M. Delitzsch, M. Deliyergiyev, A. Dell’Acqua, L. Dell’Asta, M. Dell’Orso, M. Della Pietra, D. della Volpe, M. Delmastro, P. A. Delsart, C. Deluca, D. A. DeMarco, S. Demers, M. Demichev, A. Demilly, S. P. Denisov, D. Denysiuk, D. Derendarz, J. E. Derkaoui, F. Derue, P. Dervan, K. Desch, C. Deterre, K. Dette, P. O. Deviveiros, A. Dewhurst, S. Dhaliwal, A. Di Ciaccio, L. Di Ciaccio, W. K. Di Clemente, C. Di Donato, A. Di Girolamo, B. Di Girolamo, B. Di Micco, R. Di Nardo, A. Di Simone, R. Di Sipio, D. Di Valentino, C. Diaconu, M. Diamond, F. A. Dias, M. A. Diaz, E. B. Diehl, J. Dietrich, S. Diglio, A. Dimitrievska, J. Dingfelder, P. Dita, S. Dita, F. Dittus, F. Djama, T. Djobava, J. I. Djuvsland, M. A. B. do Vale, D. Dobos, M. Dobre, C. Doglioni, T. Dohmae, J. Dolejsi, Z. Dolezal, B. A. Dolgoshein, M. Donadelli, S. Donati, P. Dondero, J. Donini, J. Dopke, A. Doria, M. T. Dova, A. T. Doyle, E. Drechsler, M. Dris, Y. Du, J. Duarte-Campderros, E. Duchovni, G. Duckeck, O. A. Ducu, D. Duda, A. Dudarev, E. M. Duffield, L. Duflot, L. Duguid, M. Dührssen, M. Dumancic, M. Dunford, H. Duran Yildiz, M. Düren, A. Durglishvili, D. Duschinger, B. Dutta, M. Dyndal, C. Eckardt, K. M. Ecker, R. C. Edgar, N. C. Edwards, T. Eifert, G. Eigen, K. Einsweiler, T. Ekelof, M. El Kacimi, V. Ellajosyula, M. Ellert, S. Elles, F. Ellinghaus, A. A. Elliot, N. Ellis, J. Elmsheuser, M. Elsing, D. Emeliyanov, Y. Enari, O. C. Endner, M. Endo, J. S. Ennis, J. Erdmann, A. Ereditato, G. Ernis, J. Ernst, M. Ernst, S. Errede, E. Ertel, M. Escalier, H. Esch, C. Escobar, B. Esposito, A. I. Etienvre, E. Etzion, H. Evans, A. Ezhilov, F. Fabbri, L. Fabbri, G. Facini, R. M. Fakhrutdinov, S. Falciano, R. J. Falla, J. Faltova, Y. Fang, M. Fanti, A. Farbin, A. Farilla, C. Farina, T. Farooque, S. Farrell, S. M. Farrington, P. Farthouat, F. Fassi, P. Fassnacht, D. Fassouliotis, M. Faucci Giannelli, A. Favareto, W. J. Fawcett, L. Fayard, O. L. Fedin, W. Fedorko, S. Feigl, L. Feligioni, C. Feng, E. J. Feng, H. Feng, A. B. Fenyuk, L. Feremenga, P. Fernandez Martinez, S. Fernandez Perez, J. Ferrando, A. Ferrari, P. Ferrari, R. Ferrari, D. E. Ferreira de Lima, A. Ferrer, D. Ferrere, C. Ferretti, A. Ferretto Parodi, F. Fiedler, A. Filipčič, M. Filipuzzi, F. Filthaut, M. Fincke-Keeler, K. D. Finelli, M. C. N. Fiolhais, L. Fiorini, A. Firan, A. Fischer, C. Fischer, J. Fischer, W. C. Fisher, N. Flaschel, I. Fleck, P. Fleischmann, G. T. Fletcher, R. R. M. Fletcher, T. Flick, A. Floderus, L. R. Flores Castillo, M. J. Flowerdew, G. T. Forcolin, A. Formica, A. Forti, A. G. Foster, D. Fournier, H. Fox, S. Fracchia, P. Francavilla, M. Franchini, D. Francis, L. Franconi, M. Franklin, M. Frate, M. Fraternali, D. Freeborn, S. M. Fressard-Batraneanu, F. Friedrich, D. Froidevaux, J. A. Frost, C. Fukunaga, E. Fullana Torregrosa, T. Fusayasu, J. Fuster, C. Gabaldon, O. Gabizon, A. Gabrielli, A. Gabrielli, G. P. Gach, S. Gadatsch, S. Gadomski, G. Gagliardi, L. G. Gagnon, P. Gagnon, C. Galea, B. Galhardo, E. J. Gallas, B. J. Gallop, P. Gallus, G. Galster, K. K. Gan, J. Gao, Y. Gao, Y. S. Gao, F. M. Garay Walls, C. García, J. E. García Navarro, M. Garcia-Sciveres, R. W. Gardner, N. Garelli, V. Garonne, A. Gascon Bravo, C. Gatti, A. Gaudiello, G. Gaudio, B. Gaur, L. Gauthier, I. L. Gavrilenko, C. Gay, G. Gaycken, E. N. Gazis, Z. Gecse, C. N. P. Gee, Ch. Geich-Gimbel, M. Geisen, M. P. Geisler, C. Gemme, M. H. Genest, C. Geng, S. Gentile, S. George, D. Gerbaudo, A. Gershon, S. Ghasemi, H. Ghazlane, M. Ghneimat, B. Giacobbe, S. Giagu, P. Giannetti, B. Gibbard, S. M. Gibson, M. Gignac, M. Gilchriese, T. P. S. Gillam, D. Gillberg, G. Gilles, D. M. Gingrich, N. Giokaris, M. P. Giordani, F. M. Giorgi, F. M. Giorgi, P. F. Giraud, P. Giromini, D. Giugni, F. Giuli, C. Giuliani, M. Giulini, B. K. Gjelsten, S. Gkaitatzis, I. Gkialas, E. L. Gkougkousis, L. K. Gladilin, C. Glasman, J. Glatzer, P. C. F. Glaysher, A. Glazov, M. Goblirsch-Kolb, J. Godlewski, S. Goldfarb, T. Golling, D. Golubkov, A. Gomes, R. Gonçalo, J. Goncalves Pinto Firmino Da Costa, G. Gonella, L. Gonella, A. Gongadze, S. González de la Hoz, G. Gonzalez Parra, S. Gonzalez-Sevilla, L. Goossens, P. A. Gorbounov, H. A. Gordon, I. Gorelov, B. Gorini, E. Gorini, A. Gorišek, E. Gornicki, A. T. Goshaw, C. Gössling, M. I. Gostkin, C. R. Goudet, D. Goujdami, A. G. Goussiou, N. Govender, E. Gozani, L. Graber, I. Grabowska-Bold, P. O. J. Gradin, P. Grafström, J. Gramling, E. Gramstad, S. Grancagnolo, V. Gratchev, P. M. Gravila, H. M. Gray, E. Graziani, Z. D. Greenwood, C. Grefe, K. Gregersen, I. M. Gregor, P. Grenier, K. Grevtsov, J. Griffiths, A. A. Grillo, K. Grimm, S. Grinstein, Ph. Gris, J.-F. Grivaz, S. Groh, J. P. Grohs, E. Gross, J. Grosse-Knetter, G. C. Grossi, Z. J. Grout, L. Guan, W. Guan, J. Guenther, F. Guescini, D. Guest, O. Gueta, E. Guido, T. Guillemin, S. Guindon, U. Gul, C. Gumpert, J. Guo, Y. Guo, S. Gupta, G. Gustavino, P. Gutierrez, N. G. Gutierrez Ortiz, C. Gutschow, C. Guyot, C. Gwenlan, C. B. Gwilliam, A. Haas, C. Haber, H. K. Hadavand, N. Haddad, A. Hadef, P. Haefner, S. Hageböck, Z. Hajduk, H. Hakobyan, M. Haleem, J. Haley, G. Halladjian, G. D. Hallewell, K. Hamacher, P. Hamal, K. Hamano, A. Hamilton, G. N. Hamity, P. G. Hamnett, L. Han, K. Hanagaki, K. Hanawa, M. Hance, B. Haney, P. Hanke, R. Hanna, J. B. Hansen, J. D. Hansen, M. C. Hansen, P. H. Hansen, K. Hara, A. S. Hard, T. Harenberg, F. Hariri, S. Harkusha, R. D. Harrington, P. F. Harrison, F. Hartjes, N. M. Hartmann, M. Hasegawa, Y. Hasegawa, A. Hasib, S. Hassani, S. Haug, R. Hauser, L. Hauswald, M. Havranek, C. M. Hawkes, R. J. Hawkings, D. Hayden, C. P. Hays, J. M. Hays, H. S. Hayward, S. J. Haywood, S. J. Head, T. Heck, V. Hedberg, L. Heelan, S. Heim, T. Heim, B. Heinemann, J. J. Heinrich, L. Heinrich, C. Heinz, J. Hejbal, L. Helary, S. Hellman, C. Helsens, J. Henderson, R. C. W. Henderson, Y. Heng, S. Henkelmann, A. M. Henriques Correia, S. Henrot-Versille, G. H. Herbert, Y. Hernández Jiménez, G. Herten, R. Hertenberger, L. Hervas, G. G. Hesketh, N. P. Hessey, J. W. Hetherly, R. Hickling, E. Higón-Rodriguez, E. Hill, J. C. Hill, K. H. Hiller, S. J. Hillier, I. Hinchliffe, E. Hines, R. R. Hinman, M. Hirose, D. Hirschbuehl, J. Hobbs, N. Hod, M. C. Hodgkinson, P. Hodgson, A. Hoecker, M. R. Hoeferkamp, F. Hoenig, D. Hohn, T. R. Holmes, M. Homann, T. M. Hong, B. H. Hooberman, W. H. Hopkins, Y. Horii, A. J. Horton, J-Y. Hostachy, S. Hou, A. Hoummada, J. Howarth, M. Hrabovsky, I. Hristova, J. Hrivnac, T. Hryn’ova, A. Hrynevich, C. Hsu, P. J. Hsu, S.-C. Hsu, D. Hu, Q. Hu, Y. Huang, Z. Hubacek, F. Hubaut, F. Huegging, T. B. Huffman, E. W. Hughes, G. Hughes, M. Huhtinen, T. A. Hülsing, P. Huo, N. Huseynov, J. Huston, J. Huth, G. Iacobucci, G. Iakovidis, I. Ibragimov, L. Iconomidou-Fayard, E. Ideal, Z. Idrissi, P. Iengo, O. Igonkina, T. Iizawa, Y. Ikegami, M. Ikeno, Y. Ilchenko, D. Iliadis, N. Ilic, T. Ince, G. Introzzi, P. Ioannou, M. Iodice, K. Iordanidou, V. Ippolito, M. Ishino, M. Ishitsuka, R. Ishmukhametov, C. Issever, S. Istin, F. Ito, J. M. Iturbe Ponce, R. Iuppa, W. Iwanski, H. Iwasaki, J. M. Izen, V. Izzo, S. Jabbar, B. Jackson, M. Jackson, P. Jackson, V. Jain, K. B. Jakobi, K. Jakobs, S. Jakobsen, T. Jakoubek, D. O. Jamin, D. K. Jana, E. Jansen, R. Jansky, J. Janssen, M. Janus, G. Jarlskog, N. Javadov, T. Javůrek, F. Jeanneau, L. Jeanty, J. Jejelava, G.-Y. Jeng, D. Jennens, P. Jenni, J. Jentzsch, C. Jeske, S. Jézéquel, H. Ji, J. Jia, H. Jiang, Y. Jiang, S. Jiggins, J. Jimenez Pena, S. Jin, A. Jinaru, O. Jinnouchi, P. Johansson, K. A. Johns, W. J. Johnson, K. Jon-And, G. Jones, R. W. L. Jones, S. Jones, T. J. Jones, J. Jongmanns, P. M. Jorge, J. Jovicevic, X. Ju, A. Juste Rozas, M. K. Köhler, A. Kaczmarska, M. Kado, H. Kagan, M. Kagan, S. J. Kahn, E. Kajomovitz, C. W. Kalderon, A. Kaluza, S. Kama, A. Kamenshchikov, N. Kanaya, S. Kaneti, L. Kanjir, V. A. Kantserov, J. Kanzaki, B. Kaplan, L. S. Kaplan, A. Kapliy, D. Kar, K. Karakostas, A. Karamaoun, N. Karastathis, M. J. Kareem, E. Karentzos, M. Karnevskiy, S. N. Karpov, Z. M. Karpova, K. Karthik, V. Kartvelishvili, A. N. Karyukhin, K. Kasahara, L. Kashif, R. D. Kass, A. Kastanas, Y. Kataoka, C. Kato, A. Katre, J. Katzy, K. Kawagoe, T. Kawamoto, G. Kawamura, S. Kazama, V. F. Kazanin, R. Keeler, R. Kehoe, J. S. Keller, J. J. Kempster, K. Kentaro, H. Keoshkerian, O. Kepka, B. P. Kerševan, S. Kersten, R. A. Keyes, F. Khalil-zada, A. Khanov, A. G. Kharlamov, T. J. Khoo, V. Khovanskiy, E. Khramov, J. Khubua, S. Kido, H. Y. Kim, S. H. Kim, Y. K. Kim, N. Kimura, O. M. Kind, B. T. King, M. King, S. B. King, J. Kirk, A. E. Kiryunin, T. Kishimoto, D. Kisielewska, F. Kiss, K. Kiuchi, O. Kivernyk, E. Kladiva, M. H. Klein, M. Klein, U. Klein, K. Kleinknecht, P. Klimek, A. Klimentov, R. Klingenberg, J. A. Klinger, T. Klioutchnikova, E.-E. Kluge, P. Kluit, S. Kluth, J. Knapik, E. Kneringer, E. B. F. G. Knoops, A. Knue, A. Kobayashi, D. Kobayashi, T. Kobayashi, M. Kobel, M. Kocian, P. Kodys, T. Koffas, E. Koffeman, T. Koi, H. Kolanoski, M. Kolb, I. Koletsou, A. A. Komar, Y. Komori, T. Kondo, N. Kondrashova, K. Köneke, A. C. König, T. Kono, R. Konoplich, N. Konstantinidis, R. Kopeliansky, S. Koperny, L. Köpke, A. K. Kopp, K. Korcyl, K. Kordas, A. Korn, A. A. Korol, I. Korolkov, E. V. Korolkova, O. Kortner, S. Kortner, T. Kosek, V. V. Kostyukhin, A. Kotwal, A. Kourkoumeli-Charalampidi, C. Kourkoumelis, V. Kouskoura, A. B. Kowalewska, R. Kowalewski, T. Z. Kowalski, C. Kozakai, W. Kozanecki, A. S. Kozhin, V. A. Kramarenko, G. Kramberger, D. Krasnopevtsev, M. W. Krasny, A. Krasznahorkay, J. K. Kraus, A. Kravchenko, M. Kretz, J. Kretzschmar, K. Kreutzfeldt, P. Krieger, K. Krizka, K. Kroeninger, H. Kroha, J. Kroll, J. Kroseberg, J. Krstic, U. Kruchonak, H. Krüger, N. Krumnack, A. Kruse, M. C. Kruse, M. Kruskal, T. Kubota, H. Kucuk, S. Kuday, J. T. Kuechler, S. Kuehn, A. Kugel, F. Kuger, A. Kuhl, T. Kuhl, V. Kukhtin, R. Kukla, Y. Kulchitsky, S. Kuleshov, M. Kuna, T. Kunigo, A. Kupco, H. Kurashige, Y. A. Kurochkin, V. Kus, E. S. Kuwertz, M. Kuze, J. Kvita, T. Kwan, D. Kyriazopoulos, A. La Rosa, J. L. La Rosa Navarro, L. La Rotonda, C. Lacasta, F. Lacava, J. Lacey, H. Lacker, D. Lacour, V. R. Lacuesta, E. Ladygin, R. Lafaye, B. Laforge, T. Lagouri, S. Lai, S. Lammers, W. Lampl, E. Lançon, U. Landgraf, M. P. J. Landon, V. S. Lang, J. C. Lange, A. J. Lankford, F. Lanni, K. Lantzsch, A. Lanza, S. Laplace, C. Lapoire, J. F. Laporte, T. Lari, F. Lasagni Manghi, M. Lassnig, P. Laurelli, W. Lavrijsen, A. T. Law, P. Laycock, T. Lazovich, M. Lazzaroni, B. Le, O. Le Dortz, E. Le Guirriec, E. P. Le Quilleuc, M. LeBlanc, T. LeCompte, F. Ledroit-Guillon, C. A. Lee, S. C. Lee, L. Lee, G. Lefebvre, M. Lefebvre, F. Legger, C. Leggett, A. Lehan, G. Lehmann Miotto, X. Lei, W. A. Leight, A. Leisos, A. G. Leister, M. A. L. Leite, R. Leitner, D. Lellouch, B. Lemmer, K. J. C. Leney, T. Lenz, B. Lenzi, R. Leone, S. Leone, C. Leonidopoulos, S. Leontsinis, G. Lerner, C. Leroy, A. A. J. Lesage, C. G. Lester, M. Levchenko, J. Levêque, D. Levin, L. J. Levinson, M. Levy, D. Lewis, A. M. Leyko, M. Leyton, B. Li, H. Li, H. L. Li, L. Li, L. Li, Q. Li, S. Li, X. Li, Y. Li, Z. Liang, B. Liberti, A. Liblong, P. Lichard, K. Lie, J. Liebal, W. Liebig, A. Limosani, S. C. Lin, T. H. Lin, B. E. Lindquist, A. E. Lionti, E. Lipeles, A. Lipniacka, M. Lisovyi, T. M. Liss, A. Lister, A. M. Litke, B. Liu, D. Liu, H. Liu, H. Liu, J. Liu, J. B. Liu, K. Liu, L. Liu, M. Liu, M. Liu, Y. L. Liu, Y. Liu, M. Livan, A. Lleres, J. Llorente Merino, S. L. Lloyd, F. Lo Sterzo, E. Lobodzinska, P. Loch, W. S. Lockman, F. K. Loebinger, A. E. Loevschall-Jensen, K. M. Loew, A. Loginov, T. Lohse, K. Lohwasser, M. Lokajicek, B. A. Long, J. D. Long, R. E. Long, L. Longo, K. A. Looper, L. Lopes, D. Lopez Mateos, B. Lopez Paredes, I. Lopez Paz, A. Lopez Solis, J. Lorenz, N. Lorenzo Martinez, M. Losada, P. J. Lösel, X. Lou, A. Lounis, J. Love, P. A. Love, H. Lu, N. Lu, H. J. Lubatti, C. Luci, A. Lucotte, C. Luedtke, F. Luehring, W. Lukas, L. Luminari, O. Lundberg, B. Lund-Jensen, P. M. Luzi, D. Lynn, R. Lysak, E. Lytken, V. Lyubushkin, H. Ma, L. L. Ma, Y. Ma, G. Maccarrone, A. Macchiolo, C. M. Macdonald, B. Maček, J. Machado Miguens, D. Madaffari, R. Madar, H. J. Maddocks, W. F. Mader, A. Madsen, J. Maeda, S. Maeland, T. Maeno, A. Maevskiy, E. Magradze, J. Mahlstedt, C. Maiani, C. Maidantchik, A. A. Maier, T. Maier, A. Maio, S. Majewski, Y. Makida, N. Makovec, B. Malaescu, Pa. Malecki, V. P. Maleev, F. Malek, U. Mallik, D. Malon, C. Malone, S. Maltezos, S. Malyukov, J. Mamuzic, G. Mancini, B. Mandelli, L. Mandelli, I. Mandić, J. Maneira, L. Manhaes de Andrade Filho, J. Manjarres Ramos, A. Mann, A. Manousos, B. Mansoulie, J. D. Mansour, R. Mantifel, M. Mantoani, S. Manzoni, L. Mapelli, G. Marceca, L. March, G. Marchiori, M. Marcisovsky, M. Marjanovic, D. E. Marley, F. Marroquim, S. P. Marsden, Z. Marshall, S. Marti-Garcia, B. Martin, T. A. Martin, V. J. Martin, B. Martin dit Latour, M. Martinez, S. Martin-Haugh, V. S. Martoiu, A. C. Martyniuk, M. Marx, A. Marzin, L. Masetti, T. Mashimo, R. Mashinistov, J. Masik, A. L. Maslennikov, I. Massa, L. Massa, P. Mastrandrea, A. Mastroberardino, T. Masubuchi, P. Mättig, J. Mattmann, J. Maurer, S. J. Maxfield, D. A. Maximov, R. Mazini, S. M. Mazza, N. C. Mc Fadden, G. Mc Goldrick, S. P. Mc Kee, A. McCarn, R. L. McCarthy, T. G. McCarthy, L. I. McClymont, E. F. McDonald, K. W. McFarlane, J. A. Mcfayden, G. Mchedlidze, S. J. McMahon, R. A. McPherson, M. Medinnis, S. Meehan, S. Mehlhase, A. Mehta, K. Meier, C. Meineck, B. Meirose, D. Melini, B. R. Mellado Garcia, M. Melo, F. Meloni, A. Mengarelli, S. Menke, E. Meoni, S. Mergelmeyer, P. Mermod, L. Merola, C. Meroni, F. S. Merritt, A. Messina, J. Metcalfe, A. S. Mete, C. Meyer, C. Meyer, J-P. Meyer, J. Meyer, H. Meyer Zu Theenhausen, F. Miano, R. P. Middleton, S. Miglioranzi, L. Mijović, G. Mikenberg, M. Mikestikova, M. Mikuž, M. Milesi, A. Milic, D. W. Miller, C. Mills, A. Milov, D. A. Milstead, A. A. Minaenko, Y. Minami, I. A. Minashvili, A. I. Mincer, B. Mindur, M. Mineev, Y. Ming, L. M. Mir, K. P. Mistry, T. Mitani, J. Mitrevski, V. A. Mitsou, A. Miucci, P. S. Miyagawa, J. U. Mjörnmark, T. Moa, K. Mochizuki, S. Mohapatra, S. Molander, R. Moles-Valls, R. Monden, M. C. Mondragon, K. Mönig, J. Monk, E. Monnier, A. Montalbano, J. Montejo Berlingen, F. Monticelli, S. Monzani, R. W. Moore, N. Morange, D. Moreno, M. Moreno Llácer, P. Morettini, D. Mori, T. Mori, M. Morii, M. Morinaga, V. Morisbak, S. Moritz, A. K. Morley, G. Mornacchi, J. D. Morris, S. S. Mortensen, L. Morvaj, M. Mosidze, J. Moss, K. Motohashi, R. Mount, E. Mountricha, S. V. Mouraviev, E. J. W. Moyse, S. Muanza, R. D. Mudd, F. Mueller, J. Mueller, R. S. P. Mueller, T. Mueller, D. Muenstermann, P. Mullen, G. A. Mullier, F. J. Munoz Sanchez, J. A. Murillo Quijada, W. J. Murray, H. Musheghyan, M. Muškinja, A. G. Myagkov, M. Myska, B. P. Nachman, O. Nackenhorst, K. Nagai, R. Nagai, K. Nagano, Y. Nagasaka, K. Nagata, M. Nagel, E. Nagy, A. M. Nairz, Y. Nakahama, K. Nakamura, T. Nakamura, I. Nakano, H. Namasivayam, R. F. Naranjo Garcia, R. Narayan, D. I. Narrias Villar, I. Naryshkin, T. Naumann, G. Navarro, R. Nayyar, H. A. Neal, P. Yu. Nechaeva, T. J. Neep, P. D. Nef, A. Negri, M. Negrini, S. Nektarijevic, C. Nellist, A. Nelson, S. Nemecek, P. Nemethy, A. A. Nepomuceno, M. Nessi, M. S. Neubauer, M. Neumann, R. M. Neves, P. Nevski, P. R. Newman, D. H. Nguyen, T. Nguyen Manh, R. B. Nickerson, R. Nicolaidou, J. Nielsen, A. Nikiforov, V. Nikolaenko, I. Nikolic-Audit, K. Nikolopoulos, J. K. Nilsen, P. Nilsson, Y. Ninomiya, A. Nisati, R. Nisius, T. Nobe, L. Nodulman, M. Nomachi, I. Nomidis, T. Nooney, S. Norberg, M. Nordberg, N. Norjoharuddeen, O. Novgorodova, S. Nowak, M. Nozaki, L. Nozka, K. Ntekas, E. Nurse, F. Nuti, F. O’grady, D. C. O’Neil, A. A. O’Rourke, V. O’Shea, F. G. Oakham, H. Oberlack, T. Obermann, J. Ocariz, A. Ochi, I. Ochoa, J. P. Ochoa-Ricoux, S. Oda, S. Odaka, H. Ogren, A. Oh, S. H. Oh, C. C. Ohm, H. Ohman, H. Oide, H. Okawa, Y. Okumura, T. Okuyama, A. Olariu, L. F. Oleiro Seabra, S. A. Olivares Pino, D. Oliveira Damazio, A. Olszewski, J. Olszowska, A. Onofre, K. Onogi, P. U. E. Onyisi, M. J. Oreglia, Y. Oren, D. Orestano, N. Orlando, R. S. Orr, B. Osculati, R. Ospanov, G. Otero y Garzon, H. Otono, M. Ouchrif, F. Ould-Saada, A. Ouraou, K. P. Oussoren, Q. Ouyang, M. Owen, R. E. Owen, V. E. Ozcan, N. Ozturk, K. Pachal, A. Pacheco Pages, C. Padilla Aranda, M. Pagáčová, S. Pagan Griso, F. Paige, P. Pais, K. Pajchel, G. Palacino, S. Palestini, M. Palka, D. Pallin, A. Palma, E. St. Panagiotopoulou, C. E. Pandini, J. G. Panduro Vazquez, P. Pani, S. Panitkin, D. Pantea, L. Paolozzi, Th. D. Papadopoulou, K. Papageorgiou, A. Paramonov, D. Paredes Hernandez, A. J. Parker, M. A. Parker, K. A. Parker, F. Parodi, J. A. Parsons, U. Parzefall, V. R. Pascuzzi, E. Pasqualucci, S. Passaggio, Fr. Pastore, G. Pásztor, S. Pataraia, J. R. Pater, T. Pauly, J. Pearce, B. Pearson, L. E. Pedersen, M. Pedersen, S. Pedraza Lopez, R. Pedro, S. V. Peleganchuk, D. Pelikan, O. Penc, C. Peng, H. Peng, J. Penwell, B. S. Peralva, M. M. Perego, D. V. Perepelitsa, E. Perez Codina, L. Perini, H. Pernegger, S. Perrella, R. Peschke, V. D. Peshekhonov, K. Peters, R. F. Y. Peters, B. A. Petersen, T. C. Petersen, E. Petit, A. Petridis, C. Petridou, P. Petroff, E. Petrolo, M. Petrov, F. Petrucci, N. E. Pettersson, A. Peyaud, R. Pezoa, P. W. Phillips, G. Piacquadio, E. Pianori, A. Picazio, E. Piccaro, M. Piccinini, M. A. Pickering, R. Piegaia, J. E. Pilcher, A. D. Pilkington, A. W. J. Pin, M. Pinamonti, J. L. Pinfold, A. Pingel, S. Pires, H. Pirumov, M. Pitt, L. Plazak, M.-A. Pleier, V. Pleskot, E. Plotnikova, P. Plucinski, D. Pluth, R. Poettgen, L. Poggioli, D. Pohl, G. Polesello, A. Poley, A. Policicchio, R. Polifka, A. Polini, C. S. Pollard, V. Polychronakos, K. Pommès, L. Pontecorvo, B. G. Pope, G. A. Popeneciu, D. S. Popovic, A. Poppleton, S. Pospisil, K. Potamianos, I. N. Potrap, C. J. Potter, C. T. Potter, G. Poulard, J. Poveda, V. Pozdnyakov, M. E. Pozo Astigarraga, P. Pralavorio, A. Pranko, S. Prell, D. Price, L. E. Price, M. Primavera, S. Prince, M. Proissl, K. Prokofiev, F. Prokoshin, S. Protopopescu, J. Proudfoot, M. Przybycien, D. Puddu, M. Purohit, P. Puzo, J. Qian, G. Qin, Y. Qin, A. Quadt, W. B. Quayle, M. Queitsch-Maitland, D. Quilty, S. Raddum, V. Radeka, V. Radescu, S. K. Radhakrishnan, P. Radloff, P. Rados, F. Ragusa, G. Rahal, J. A. Raine, S. Rajagopalan, M. Rammensee, C. Rangel-Smith, M. G. Ratti, F. Rauscher, S. Rave, T. Ravenscroft, I. Ravinovich, M. Raymond, A. L. Read, N. P. Readioff, M. Reale, D. M. Rebuzzi, A. Redelbach, G. Redlinger, R. Reece, K. Reeves, L. Rehnisch, J. Reichert, H. Reisin, C. Rembser, H. Ren, M. Rescigno, S. Resconi, O. L. Rezanova, P. Reznicek, R. Rezvani, R. Richter, S. Richter, E. Richter-Was, O. Ricken, M. Ridel, P. Rieck, C. J. Riegel, J. Rieger, O. Rifki, M. Rijssenbeek, A. Rimoldi, M. Rimoldi, L. Rinaldi, B. Ristić, E. Ritsch, I. Riu, F. Rizatdinova, E. Rizvi, C. Rizzi, S. H. Robertson, A. Robichaud-Veronneau, D. Robinson, J. E. M. Robinson, A. Robson, C. Roda, Y. Rodina, A. Rodriguez Perez, D. Rodriguez Rodriguez, S. Roe, C. S. Rogan, O. Røhne, A. Romaniouk, M. Romano, S. M. Romano Saez, E. Romero Adam, N. Rompotis, M. Ronzani, L. Roos, E. Ros, S. Rosati, K. Rosbach, P. Rose, O. Rosenthal, N.-A. Rosien, V. Rossetti, E. Rossi, L. P. Rossi, J. H. N. Rosten, R. Rosten, M. Rotaru, I. Roth, J. Rothberg, D. Rousseau, C. R. Royon, A. Rozanov, Y. Rozen, X. Ruan, F. Rubbo, M. S. Rudolph, F. Rühr, A. Ruiz-Martinez, Z. Rurikova, N. A. Rusakovich, A. Ruschke, H. L. Russell, J. P. Rutherfoord, N. Ruthmann, Y. F. Ryabov, M. Rybar, G. Rybkin, S. Ryu, A. Ryzhov, G. F. Rzehorz, A. F. Saavedra, G. Sabato, S. Sacerdoti, H. F-W. Sadrozinski, R. Sadykov, F. Safai Tehrani, P. Saha, M. Sahinsoy, M. Saimpert, T. Saito, H. Sakamoto, Y. Sakurai, G. Salamanna, A. Salamon, J. E. Salazar Loyola, D. Salek, P. H. Sales De Bruin, D. Salihagic, A. Salnikov, J. Salt, D. Salvatore, F. Salvatore, A. Salvucci, A. Salzburger, D. Sammel, D. Sampsonidis, A. Sanchez, J. Sánchez, V. Sanchez Martinez, H. Sandaker, R. L. Sandbach, H. G. Sander, M. Sandhoff, C. Sandoval, R. Sandstroem, D. P. C. Sankey, M. Sannino, A. Sansoni, C. Santoni, R. Santonico, H. Santos, I. Santoyo Castillo, K. Sapp, A. Sapronov, J. G. Saraiva, B. Sarrazin, O. Sasaki, Y. Sasaki, K. Sato, G. Sauvage, E. Sauvan, G. Savage, P. Savard, C. Sawyer, L. Sawyer, J. Saxon, C. Sbarra, A. Sbrizzi, T. Scanlon, D. A. Scannicchio, M. Scarcella, V. Scarfone, J. Schaarschmidt, P. Schacht, B. M. Schachtner, D. Schaefer, R. Schaefer, J. Schaeffer, S. Schaepe, S. Schaetzel, U. Schäfer, A. C. Schaffer, D. Schaile, R. D. Schamberger, V. Scharf, V. A. Schegelsky, D. Scheirich, M. Schernau, C. Schiavi, S. Schier, C. Schillo, M. Schioppa, S. Schlenker, K. R. Schmidt-Sommerfeld, K. Schmieden, C. Schmitt, S. Schmitt, S. Schmitz, B. Schneider, U. Schnoor, L. Schoeffel, A. Schoening, B. D. Schoenrock, E. Schopf, M. Schott, J. Schovancova, S. Schramm, M. Schreyer, N. Schuh, M. J. Schultens, H.-C. Schultz-Coulon, H. Schulz, M. Schumacher, B. A. Schumm, Ph. Schune, A. Schwartzman, T. A. Schwarz, Ph. Schwegler, H. Schweiger, Ph. Schwemling, R. Schwienhorst, J. Schwindling, T. Schwindt, G. Sciolla, F. Scuri, F. Scutti, J. Searcy, P. Seema, S. C. Seidel, A. Seiden, F. Seifert, J. M. Seixas, G. Sekhniaidze, K. Sekhon, S. J. Sekula, D. M. Seliverstov, N. Semprini-Cesari, C. Serfon, L. Serin, L. Serkin, M. Sessa, R. Seuster, H. Severini, T. Sfiligoj, F. Sforza, A. Sfyrla, E. Shabalina, N. W. Shaikh, L. Y. Shan, R. Shang, J. T. Shank, M. Shapiro, P. B. Shatalov, K. Shaw, S. M. Shaw, A. Shcherbakova, C. Y. Shehu, P. Sherwood, L. Shi, S. Shimizu, C. O. Shimmin, M. Shimojima, M. Shiyakova, A. Shmeleva, D. Shoaleh Saadi, M. J. Shochet, S. Shojaii, S. Shrestha, E. Shulga, M. A. Shupe, P. Sicho, A. M. Sickles, P. E. Sidebo, O. Sidiropoulou, D. Sidorov, A. Sidoti, F. Siegert, Dj. Sijacki, J. Silva, S. B. Silverstein, V. Simak, O. Simard, Lj. Simic, S. Simion, E. Simioni, B. Simmons, D. Simon, M. Simon, P. Sinervo, N. B. Sinev, M. Sioli, G. Siragusa, S. Yu. Sivoklokov, J. Sjölin, T. B. Sjursen, M. B. Skinner, H. P. Skottowe, P. Skubic, M. Slater, T. Slavicek, M. Slawinska, K. Sliwa, R. Slovak, V. Smakhtin, B. H. Smart, L. Smestad, J. Smiesko, S. Yu. Smirnov, Y. Smirnov, L. N. Smirnova, O. Smirnova, M. N. K. Smith, R. W. Smith, M. Smizanska, K. Smolek, A. A. Snesarev, S. Snyder, R. Sobie, F. Socher, A. Soffer, D. A. Soh, G. Sokhrannyi, C. A. Solans Sanchez, M. Solar, E. Yu. Soldatov, U. Soldevila, A. A. Solodkov, A. Soloshenko, O. V. Solovyanov, V. Solovyev, P. Sommer, H. Son, H. Y. Song, A. Sood, A. Sopczak, V. Sopko, V. Sorin, D. Sosa, C. L. Sotiropoulou, R. Soualah, A. M. Soukharev, D. South, B. C. Sowden, S. Spagnolo, M. Spalla, M. Spangenberg, F. Spanò, D. Sperlich, F. Spettel, R. Spighi, G. Spigo, L. A. Spiller, M. Spousta, R. D. St. Denis, A. Stabile, R. Stamen, S. Stamm, E. Stanecka, R. W. Stanek, C. Stanescu, M. Stanescu-Bellu, M. M. Stanitzki, S. Stapnes, E. A. Starchenko, G. H. Stark, J. Stark, P. Staroba, P. Starovoitov, S. Stärz, R. Staszewski, P. Steinberg, B. Stelzer, H. J. Stelzer, O. Stelzer-Chilton, H. Stenzel, G. A. Stewart, J. A. Stillings, M. C. Stockton, M. Stoebe, G. Stoicea, P. Stolte, S. Stonjek, A. R. Stradling, A. Straessner, M. E. Stramaglia, J. Strandberg, S. Strandberg, A. Strandlie, M. Strauss, P. Strizenec, R. Ströhmer, D. M. Strom, R. Stroynowski, A. Strubig, S. A. Stucci, B. Stugu, N. A. Styles, D. Su, J. Su, R. Subramaniam, S. Suchek, Y. Sugaya, M. Suk, V. V. Sulin, S. Sultansoy, T. Sumida, S. Sun, X. Sun, J. E. Sundermann, K. Suruliz, G. Susinno, M. R. Sutton, S. Suzuki, M. Svatos, M. Swiatlowski, I. Sykora, T. Sykora, D. Ta, C. Taccini, K. Tackmann, J. Taenzer, A. Taffard, R. Tafirout, N. Taiblum, H. Takai, R. Takashima, T. Takeshita, Y. Takubo, M. Talby, A. A. Talyshev, K. G. Tan, J. Tanaka, R. Tanaka, S. Tanaka, B. B. Tannenwald, S. Tapia Araya, S. Tapprogge, S. Tarem, G. F. Tartarelli, P. Tas, M. Tasevsky, T. Tashiro, E. Tassi, A. Tavares Delgado, Y. Tayalati, A. C. Taylor, G. N. Taylor, P. T. E. Taylor, W. Taylor, F. A. Teischinger, P. Teixeira-Dias, K. K. Temming, D. Temple, H. Ten Kate, P. K. Teng, J. J. Teoh, F. Tepel, S. Terada, K. Terashi, J. Terron, S. Terzo, M. Testa, R. J. Teuscher, T. Theveneaux-Pelzer, J. P. Thomas, J. Thomas-Wilsker, E. N. Thompson, P. D. Thompson, A. S. Thompson, L. A. Thomsen, E. Thomson, M. Thomson, M. J. Tibbetts, R. E. Ticse Torres, V. O. Tikhomirov, Yu. A. Tikhonov, S. Timoshenko, P. Tipton, S. Tisserant, K. Todome, T. Todorov, S. Todorova-Nova, J. Tojo, S. Tokár, K. Tokushuku, E. Tolley, L. Tomlinson, M. Tomoto, L. Tompkins, K. Toms, B. Tong, E. Torrence, H. Torres, E. Torró Pastor, J. Toth, F. Touchard, D. R. Tovey, T. Trefzger, A. Tricoli, I. M. Trigger, S. Trincaz-Duvoid, M. F. Tripiana, W. Trischuk, B. Trocmé, A. Trofymov, C. Troncon, M. Trottier-McDonald, M. Trovatelli, L. Truong, M. Trzebinski, A. Trzupek, J. C-L. Tseng, P. V. Tsiareshka, G. Tsipolitis, N. Tsirintanis, S. Tsiskaridze, V. Tsiskaridze, E. G. Tskhadadze, K. M. Tsui, I. I. Tsukerman, V. Tsulaia, S. Tsuno, D. Tsybychev, A. Tudorache, V. Tudorache, A. N. Tuna, S. A. Tupputi, S. Turchikhin, D. Turecek, D. Turgeman, R. Turra, A. J. Turvey, P. M. Tuts, M. Tyndel, G. Ucchielli, I. Ueda, R. Ueno, M. Ughetto, F. Ukegawa, G. Unal, A. Undrus, G. Unel, F. C. Ungaro, Y. Unno, C. Unverdorben, J. Urban, P. Urquijo, P. Urrejola, G. Usai, A. Usanova, L. Vacavant, V. Vacek, B. Vachon, C. Valderanis, E. Valdes Santurio, N. Valencic, S. Valentinetti, A. Valero, L. Valery, S. Valkar, S. Vallecorsa, J. A. Valls Ferrer, W. Van Den Wollenberg, P. C. Van Der Deijl, R. van der Geer, H. van der Graaf, N. van Eldik, P. van Gemmeren, J. Van Nieuwkoop, I. van Vulpen, M. C. van Woerden, M. Vanadia, W. Vandelli, R. Vanguri, A. Vaniachine, P. Vankov, G. Vardanyan, R. Vari, E. W. Varnes, T. Varol, D. Varouchas, A. Vartapetian, K. E. Varvell, J. G. Vasquez, F. Vazeille, T. Vazquez Schroeder, J. Veatch, L. M. Veloce, F. Veloso, S. Veneziano, A. Ventura, M. Venturi, N. Venturi, A. Venturini, V. Vercesi, M. Verducci, W. Verkerke, J. C. Vermeulen, A. Vest, M. C. Vetterli, O. Viazlo, I. Vichou, T. Vickey, O. E. Vickey Boeriu, G. H. A. Viehhauser, S. Viel, L. Vigani, R. Vigne, M. Villa, M. Villaplana Perez, E. Vilucchi, M. G. Vincter, V. B. Vinogradov, C. Vittori, I. Vivarelli, S. Vlachos, M. Vlasak, M. Vogel, P. Vokac, G. Volpi, M. Volpi, H. von der Schmitt, E. von Toerne, V. Vorobel, K. Vorobev, M. Vos, R. Voss, J. H. Vossebeld, N. Vranjes, M. Vranjes Milosavljevic, V. Vrba, M. Vreeswijk, R. Vuillermet, I. Vukotic, Z. Vykydal, P. Wagner, W. Wagner, H. Wahlberg, S. Wahrmund, J. Wakabayashi, J. Walder, R. Walker, W. Walkowiak, V. Wallangen, C. Wang, C. Wang, F. Wang, H. Wang, H. Wang, J. Wang, J. Wang, K. Wang, R. Wang, S. M. Wang, T. Wang, T. Wang, W. Wang, X. Wang, C. Wanotayaroj, A. Warburton, C. P. Ward, D. R. Wardrope, A. Washbrook, P. M. Watkins, A. T. Watson, M. F. Watson, G. Watts, S. Watts, B. M. Waugh, S. Webb, M. S. Weber, S. W. Weber, J. S. Webster, A. R. Weidberg, B. Weinert, J. Weingarten, C. Weiser, H. Weits, P. S. Wells, T. Wenaus, T. Wengler, S. Wenig, N. Wermes, M. Werner, P. Werner, M. Wessels, J. Wetter, K. Whalen, N. L. Whallon, A. M. Wharton, A. White, M. J. White, R. White, D. Whiteson, F. J. Wickens, W. Wiedenmann, M. Wielers, P. Wienemann, C. Wiglesworth, L. A. M. Wiik-Fuchs, A. Wildauer, F. Wilk, H. G. Wilkens, H. H. Williams, S. Williams, C. Willis, S. Willocq, J. A. Wilson, I. Wingerter-Seez, F. Winklmeier, O. J. Winston, B. T. Winter, M. Wittgen, J. Wittkowski, S. J. Wollstadt, M. W. Wolter, H. Wolters, B. K. Wosiek, J. Wotschack, M. J. Woudstra, K. W. Wozniak, M. Wu, M. Wu, S. L. Wu, X. Wu, Y. Wu, T. R. Wyatt, B. M. Wynne, S. Xella, D. Xu, L. Xu, B. Yabsley, S. Yacoob, R. Yakabe, D. Yamaguchi, Y. Yamaguchi, A. Yamamoto, S. Yamamoto, T. Yamanaka, K. Yamauchi, Y. Yamazaki, Z. Yan, H. Yang, H. Yang, Y. Yang, Z. Yang, W-M. Yao, Y. C. Yap, Y. Yasu, E. Yatsenko, K. H. Yau Wong, J. Ye, S. Ye, I. Yeletskikh, A. L. Yen, E. Yildirim, K. Yorita, R. Yoshida, K. Yoshihara, C. Young, C. J. S. Young, S. Youssef, D. R. Yu, J. Yu, J. M. Yu, J. Yu, L. Yuan, S. P. Y. Yuen, I. Yusuff, B. Zabinski, R. Zaidan, A. M. Zaitsev, N. Zakharchuk, J. Zalieckas, A. Zaman, S. Zambito, L. Zanello, D. Zanzi, C. Zeitnitz, M. Zeman, A. Zemla, J. C. Zeng, Q. Zeng, K. Zengel, O. Zenin, T. Ženiš, D. Zerwas, D. Zhang, F. Zhang, G. Zhang, H. Zhang, J. Zhang, L. Zhang, R. Zhang, R. Zhang, X. Zhang, Z. Zhang, X. Zhao, Y. Zhao, Z. Zhao, A. Zhemchugov, J. Zhong, B. Zhou, C. Zhou, L. Zhou, L. Zhou, M. Zhou, N. Zhou, C. G. Zhu, H. Zhu, J. Zhu, Y. Zhu, X. Zhuang, K. Zhukov, A. Zibell, D. Zieminska, N. I. Zimine, C. Zimmermann, S. Zimmermann, Z. Zinonos, M. Zinser, M. Ziolkowski, L. Živković, G. Zobernig, A. Zoccoli, M. zur Nedden, G. Zurzolo, L. Zwalinski

**Affiliations:** 1 Department of Physics, University of Adelaide, Adelaide, Australia; 2 Physics Department, SUNY Albany, Albany, NY USA; 3 Department of Physics, University of Alberta, Edmonton, AB Canada; 4 Department of Physics, Ankara University, Ankara, Turkey; 5 Istanbul Aydin University, Istanbul, Turkey; 6 Division of Physics, TOBB University of Economics and Technology, Ankara, Turkey; 7 LAPP, CNRS/IN2P3 and Université Savoie Mont Blanc, Annecy-le-Vieux, France; 8 High Energy Physics Division, Argonne National Laboratory, Argonne, IL USA; 9 Department of Physics, University of Arizona, Tucson, AZ USA; 10 Department of Physics, The University of Texas at Arlington, Arlington, TX USA; 11 Physics Department, University of Athens, Athens, Greece; 12 Physics Department, National Technical University of Athens, Zografou, Greece; 13 Department of Physics, The University of Texas at Austin, Austin, TX USA; 14 Institute of Physics, Azerbaijan Academy of Sciences, Baku, Azerbaijan; 15 Institut de Física d’Altes Energies (IFAE), The Barcelona Institute of Science and Technology, Barcelona, Spain; 16 Institute of Physics, University of Belgrade, Belgrade, Serbia; 17 Department for Physics and Technology, University of Bergen, Bergen, Norway; 18 Physics Division, Lawrence Berkeley National Laboratory and University of California, Berkeley, CA USA; 19 Department of Physics, Humboldt University, Berlin, Germany; 20 Albert Einstein Center for Fundamental Physics and Laboratory for High Energy Physics, University of Bern, Bern, Switzerland; 21 School of Physics and Astronomy, University of Birmingham, Birmingham, UK; 22 Department of Physics, Bogazici University, Istanbul, Turkey; 23 Department of Physics Engineering, Gaziantep University, Gaziantep, Turkey; 24 Faculty of Engineering and Natural Sciences, Istanbul Bilgi University, Istanbul, Turkey; 25 Faculty of Engineering and Natural Sciences, Bahcesehir University, Istanbul, Turkey; 26 Centro de Investigaciones, Universidad Antonio Narino, Bogotá, Colombia; 27 INFN Sezione di Bologna, Bologna, Italy; 28 Dipartimento di Fisica e Astronomia, Università di Bologna, Bologna, Italy; 29 Physikalisches Institut, University of Bonn, Bonn, Germany; 30 Department of Physics, Boston University, Boston, MA USA; 31 Department of Physics, Brandeis University, Waltham, MA USA; 32 Universidade Federal do Rio De Janeiro COPPE/EE/IF, Rio de Janeiro, Brazil; 33 Electrical Circuits Department, Federal University of Juiz de Fora (UFJF), Juiz de Fora, Brazil; 34 Federal University of Sao Joao del Rei (UFSJ), Sao Joao del Rei, Brazil; 35 Instituto de Fisica, Universidade de Sao Paulo, São Paulo, Brazil; 36 Physics Department, Brookhaven National Laboratory, Upton, NY USA; 37 Transilvania University of Brasov, Brasov, Romania; 38 National Institute of Physics and Nuclear Engineering, Bucharest, Romania; 39 Physics Department, National Institute for Research and Development of Isotopic and Molecular Technologies, Cluj Napoca, Romania; 40 University Politehnica Bucharest, Bucharest, Romania; 41 West University in Timisoara, Timisoara, Romania; 42 Departamento de Física, Universidad de Buenos Aires, Buenos Aires, Argentina; 43 Cavendish Laboratory, University of Cambridge, Cambridge, UK; 44 Department of Physics, Carleton University, Ottawa, ON Canada; 45 CERN, Geneva, Switzerland; 46 Enrico Fermi Institute, University of Chicago, Chicago, IL USA; 47 Departamento de Física, Pontificia Universidad Católica de Chile, Santiago, Chile; 48 Departamento de Física, Universidad Técnica Federico Santa María, Valparaiso, Chile; 49 Institute of High Energy Physics, Chinese Academy of Sciences, Beijing, China; 50 Department of Modern Physics, University of Science and Technology of China, Hefei, Anhui China; 51 Department of Physics, Nanjing University, Nanjing, Jiangsu China; 52 School of Physics, Shandong University, Jinan, Shandong China; 53 Shanghai Key Laboratory for Particle Physics and Cosmology, Department of Physics and Astronomy, Shanghai Jiao Tong University, (also affiliated with PKU-CHEP), Shanghai, China; 54 Physics Department, Tsinghua University, Beijing, 100084 China; 55 Laboratoire de Physique Corpusculaire, Clermont Université and Université Blaise Pascal and CNRS/IN2P3, Clermont-Ferrand, France; 56 Nevis Laboratory, Columbia University, Irvington, NY USA; 57 Niels Bohr Institute, University of Copenhagen, Kobenhavn, Denmark; 58 INFN Gruppo Collegato di Cosenza, Laboratori Nazionali di Frascati, Frascati, Italy; 59 Dipartimento di Fisica, Università della Calabria, Rende, Italy; 60 Faculty of Physics and Applied Computer Science, AGH University of Science and Technology, Krakow, Poland; 61 Marian Smoluchowski Institute of Physics, Jagiellonian University, Krakow, Poland; 62 Institute of Nuclear Physics, Polish Academy of Sciences, Krakow, Poland; 63 Physics Department, Southern Methodist University, Dallas, TX USA; 64 Physics Department, University of Texas at Dallas, Richardson, TX USA; 65 DESY, Hamburg and Zeuthen, Germany; 66 Institut für Experimentelle Physik IV, Technische Universität Dortmund, Dortmund, Germany; 67 Institut für Kern- und Teilchenphysik, Technische Universität Dresden, Dresden, Germany; 68 Department of Physics, Duke University, Durham, NC USA; 69 SUPA-School of Physics and Astronomy, University of Edinburgh, Edinburgh, UK; 70 INFN Laboratori Nazionali di Frascati, Frascati, Italy; 71 Fakultät für Mathematik und Physik, Albert-Ludwigs-Universität, Freiburg, Germany; 72 Section de Physique, Université de Genève, Geneva, Switzerland; 73 INFN Sezione di Genova, Genoa, Italy; 74 Dipartimento di Fisica, Università di Genova, Genoa, Italy; 75 E. Andronikashvili Institute of Physics, Iv. Javakhishvili Tbilisi State University, Tbilisi, Georgia; 76 High Energy Physics Institute, Tbilisi State University, Tbilisi, Georgia; 77 II Physikalisches Institut, Justus-Liebig-Universität Giessen, Giessen, Germany; 78 SUPA-School of Physics and Astronomy, University of Glasgow, Glasgow, UK; 79 II Physikalisches Institut, Georg-August-Universität, Göttingen, Germany; 80 Laboratoire de Physique Subatomique et de Cosmologie, Université Grenoble-Alpes, CNRS/IN2P3, Grenoble, France; 81 Department of Physics, Hampton University, Hampton, VA USA; 82 Laboratory for Particle Physics and Cosmology, Harvard University, Cambridge, MA USA; 83 Kirchhoff-Institut für Physik, Ruprecht-Karls-Universität Heidelberg, Heidelberg, Germany; 84 Physikalisches Institut, Ruprecht-Karls-Universität Heidelberg, Heidelberg, Germany; 85 ZITI Institut für technische Informatik, Ruprecht-Karls-Universität Heidelberg, Mannheim, Germany; 86 Faculty of Applied Information Science, Hiroshima Institute of Technology, Hiroshima, Japan; 87 Department of Physics, The Chinese University of Hong Kong, Shatin, NT Hong Kong; 88 Department of Physics, The University of Hong Kong, Hong Kong, China; 89 Department of Physics, The Hong Kong University of Science and Technology, Clear Water Bay, Kowloon, Hong Kong China; 90 Department of Physics, Indiana University, Bloomington, IN USA; 91 Institut für Astro- und Teilchenphysik, Leopold-Franzens-Universität, Innsbruck, Austria; 92 University of Iowa, Iowa City, IA USA; 93 Department of Physics and Astronomy, Iowa State University, Ames, IA USA; 94 Joint Institute for Nuclear Research, JINR Dubna, Dubna, Russia; 95 KEK, High Energy Accelerator Research Organization, Tsukuba, Japan; 96 Graduate School of Science, Kobe University, Kobe, Japan; 97 Faculty of Science, Kyoto University, Kyoto, Japan; 98 Kyoto University of Education, Kyoto, Japan; 99 Department of Physics, Kyushu University, Fukuoka, Japan; 100 Instituto de Física La Plata, Universidad Nacional de La Plata and CONICET, La Plata, Argentina; 101 Physics Department, Lancaster University, Lancaster, UK; 102 INFN Sezione di Lecce, Lecce, Italy; 103 Dipartimento di Matematica e Fisica, Università del Salento, Lecce, Italy; 104 Oliver Lodge Laboratory, University of Liverpool, Liverpool, UK; 105 Department of Physics, Jožef Stefan Institute and University of Ljubljana, Ljubljana, Slovenia; 106 School of Physics and Astronomy, Queen Mary University of London, London, UK; 107 Department of Physics, Royal Holloway University of London, Surrey, UK; 108 Department of Physics and Astronomy, University College London, London, UK; 109 Louisiana Tech University, Ruston, LA USA; 110 Laboratoire de Physique Nucléaire et de Hautes Energies, UPMC and Université Paris-Diderot and CNRS/IN2P3, Paris, France; 111 Fysiska institutionen, Lunds universitet, Lund, Sweden; 112 Departamento de Fisica Teorica C-15, Universidad Autonoma de Madrid, Madrid, Spain; 113 Institut für Physik, Universität Mainz, Mainz, Germany; 114 School of Physics and Astronomy, University of Manchester, Manchester, UK; 115 CPPM, Aix-Marseille Université and CNRS/IN2P3, Marseille, France; 116 Department of Physics, University of Massachusetts, Amherst, MA USA; 117 Department of Physics, McGill University, Montreal, QC Canada; 118 School of Physics, University of Melbourne, Melbourne, Victoria Australia; 119 Department of Physics, The University of Michigan, Ann Arbor, MI USA; 120 Department of Physics and Astronomy, Michigan State University, East Lansing, MI USA; 121 INFN Sezione di Milano, Milan, Italy; 122 Dipartimento di Fisica, Università di Milano, Milan, Italy; 123 B.I. Stepanov Institute of Physics, National Academy of Sciences of Belarus, Minsk, Republic of Belarus; 124 National Scientific and Educational Centre for Particle and High Energy Physics, Minsk, Republic of Belarus; 125 Group of Particle Physics, University of Montreal, Montreal, QC Canada; 126 P.N. Lebedev Physical Institute of the Russian Academy of Sciences, Moscow, Russia; 127 Institute for Theoretical and Experimental Physics (ITEP), Moscow, Russia; 128 National Research Nuclear University MEPhI, Moscow, Russia; 129 D.V. Skobeltsyn Institute of Nuclear Physics, M.V. Lomonosov Moscow State University, Moscow, Russia; 130 Fakultät für Physik, Ludwig-Maximilians-Universität München, Munich, Germany; 131 Max-Planck-Institut für Physik (Werner-Heisenberg-Institut), Munich, Germany; 132 Nagasaki Institute of Applied Science, Nagasaki, Japan; 133 Graduate School of Science and Kobayashi-Maskawa Institute, Nagoya University, Nagoya, Japan; 134 INFN Sezione di Napoli, Naples, Italy; 135 Dipartimento di Fisica, Università di Napoli, Naples, Italy; 136 Department of Physics and Astronomy, University of New Mexico, Albuquerque, NM USA; 137 Institute for Mathematics, Astrophysics and Particle Physics, Radboud University Nijmegen/Nikhef, Nijmegen, The Netherlands; 138 Nikhef National Institute for Subatomic Physics and University of Amsterdam, Amsterdam, The Netherlands; 139 Department of Physics, Northern Illinois University, DeKalb, IL USA; 140 Budker Institute of Nuclear Physics, SB RAS, Novosibirsk, Russia; 141 Department of Physics, New York University, New York, NY USA; 142 Ohio State University, Columbus, OH USA; 143 Faculty of Science, Okayama University, Okayama, Japan; 144 Homer L. Dodge Department of Physics and Astronomy, University of Oklahoma, Norman, OK USA; 145 Department of Physics, Oklahoma State University, Stillwater, OK USA; 146 Palacký University, RCPTM, Olomouc, Czech Republic; 147 Center for High Energy Physics, University of Oregon, Eugene, OR USA; 148 LAL, Univ. Paris-Sud, CNRS/IN2P3, Université Paris Saclay, Orsay, France; 149 Graduate School of Science, Osaka University, Osaka, Japan; 150 Department of Physics, University of Oslo, Oslo, Norway; 151 Department of Physics, Oxford University, Oxford, UK; 152 INFN Sezione di Pavia, Pavia, Italy; 153 Dipartimento di Fisica, Università di Pavia, Pavia, Italy; 154 Department of Physics, University of Pennsylvania, Philadelphia, PA USA; 155 National Research Centre “Kurchatov Institute” B.P.Konstantinov Petersburg Nuclear Physics Institute, St. Petersburg, Russia; 156 INFN Sezione di Pisa, Pisa, Italy; 157 Dipartimento di Fisica E. Fermi, Università di Pisa, Pisa, Italy; 158 Department of Physics and Astronomy, University of Pittsburgh, Pittsburgh, PA USA; 159 Laboratório de Instrumentação e Física Experimental de Partículas-LIP, Lisbon, Portugal; 160 Faculdade de Ciências, Universidade de Lisboa, Lisbon, Portugal; 161 Department of Physics, University of Coimbra, Coimbra, Portugal; 162 Centro de Física Nuclear da Universidade de Lisboa, Lisbon, Portugal; 163 Departamento de Fisica, Universidade do Minho, Braga, Portugal; 164 Departamento de Fisica Teorica y del Cosmos and CAFPE, Universidad de Granada, Granada, Spain; 165 Dep Fisica and CEFITEC of Faculdade de Ciencias e Tecnologia, Universidade Nova de Lisboa, Caparica, Portugal; 166 Institute of Physics, Academy of Sciences of the Czech Republic, Praha, Czech Republic; 167 Czech Technical University in Prague, Praha, Czech Republic; 168 Faculty of Mathematics and Physics, Charles University in Prague, Praha, Czech Republic; 169 State Research Center Institute for High Energy Physics (Protvino), NRC KI, Protvino, Russia; 170 Particle Physics Department, Rutherford Appleton Laboratory, Didcot, UK; 171 INFN Sezione di Roma, Rome, Italy; 172 Dipartimento di Fisica, Sapienza Università di Roma, Rome, Italy; 173 INFN Sezione di Roma Tor Vergata, Rome, Italy; 174 Dipartimento di Fisica, Università di Roma Tor Vergata, Rome, Italy; 175 INFN Sezione di Roma Tre, Rome, Italy; 176 Dipartimento di Matematica e Fisica, Università Roma Tre, Rome, Italy; 177 Faculté des Sciences Ain Chock, Réseau Universitaire de Physique des Hautes Energies-Université Hassan II, Casablanca, Morocco; 178 Centre National de l’Energie des Sciences Techniques Nucleaires, Rabat, Morocco; 179 Faculté des Sciences Semlalia, Université Cadi Ayyad, LPHEA-Marrakech, Marrakech, Morocco; 180 Faculté des Sciences, Université Mohamed Premier and LPTPM, Oujda, Morocco; 181 Faculté des Sciences, Université Mohammed V, Rabat, Morocco; 182 DSM/IRFU (Institut de Recherches sur les Lois Fondamentales de l’Univers), CEA Saclay (Commissariat à l’Energie Atomique et aux Energies Alternatives), Gif-sur-Yvette, France; 183 Santa Cruz Institute for Particle Physics, University of California Santa Cruz, Santa Cruz, CA USA; 184 Department of Physics, University of Washington, Seattle, WA USA; 185 Department of Physics and Astronomy, University of Sheffield, Sheffield, UK; 186 Department of Physics, Shinshu University, Nagano, Japan; 187 Fachbereich Physik, Universität Siegen, Siegen, Germany; 188 Department of Physics, Simon Fraser University, Burnaby, BC Canada; 189 SLAC National Accelerator Laboratory, Stanford, CA USA; 190 Faculty of Mathematics, Physics and Informatics, Comenius University, Bratislava, Slovak Republic; 191 Department of Subnuclear Physics, Institute of Experimental Physics of the Slovak Academy of Sciences, Kosice, Slovak Republic; 192 Department of Physics, University of Cape Town, Cape Town, South Africa; 193 Department of Physics, University of Johannesburg, Johannesburg, South Africa; 194 School of Physics, University of the Witwatersrand, Johannesburg, South Africa; 195 Department of Physics, Stockholm University, Stockholm, Sweden; 196 The Oskar Klein Centre, Stockholm, Sweden; 197 Physics Department, Royal Institute of Technology, Stockholm, Sweden; 198 Departments of Physics and Astronomy and Chemistry, Stony Brook University, Stony Brook, NY USA; 199 Department of Physics and Astronomy, University of Sussex, Brighton, UK; 200 School of Physics, University of Sydney, Sydney, Australia; 201 Institute of Physics, Academia Sinica, Taipei, Taiwan; 202 Department of Physics, Technion: Israel Institute of Technology, Haifa, Israel; 203 Raymond and Beverly Sackler School of Physics and Astronomy, Tel Aviv University, Tel Aviv, Israel; 204 Department of Physics, Aristotle University of Thessaloniki, Thessaloníki, Greece; 205 International Center for Elementary Particle Physics and Department of Physics, The University of Tokyo, Tokyo, Japan; 206 Graduate School of Science and Technology, Tokyo Metropolitan University, Tokyo, Japan; 207 Department of Physics, Tokyo Institute of Technology, Tokyo, Japan; 208 Department of Physics, University of Toronto, Toronto, ON Canada; 209 TRIUMF, Vancouver, BC Canada; 210 Department of Physics and Astronomy, York University, Toronto, ON Canada; 211 Faculty of Pure and Applied Sciences, and Center for Integrated Research in Fundamental Science and Engineering, University of Tsukuba, Tsukuba, Japan; 212 Department of Physics and Astronomy, Tufts University, Medford, MA USA; 213 Department of Physics and Astronomy, University of California Irvine, Irvine, CA USA; 214 INFN Gruppo Collegato di Udine, Sezione di Trieste, Udine, Italy; 215 ICTP, Trieste, Italy; 216 Dipartimento di Chimica Fisica e Ambiente, Università di Udine, Udine, Italy; 217 Department of Physics and Astronomy, University of Uppsala, Uppsala, Sweden; 218 Department of Physics, University of Illinois, Urbana, IL USA; 219 Instituto de Fisica Corpuscular (IFIC) and Departamento de Fisica Atomica, Molecular y Nuclear and Departamento de Ingeniería Electrónica and Instituto de Microelectrónica de Barcelona (IMB-CNM), University of Valencia and CSIC, Valencia, Spain; 220 Department of Physics, University of British Columbia, Vancouver, BC Canada; 221 Department of Physics and Astronomy, University of Victoria, Victoria, BC Canada; 222 Department of Physics, University of Warwick, Coventry, UK; 223 Waseda University, Tokyo, Japan; 224 Department of Particle Physics, The Weizmann Institute of Science, Rehovot, Israel; 225 Department of Physics, University of Wisconsin, Madison, WI USA; 226 Fakultät für Physik und Astronomie, Julius-Maximilians-Universität, Würzburg, Germany; 227 Fakultät für Mathematik und Naturwissenschaften, Fachgruppe Physik, Bergische Universität Wuppertal, Wuppertal, Germany; 228 Department of Physics, Yale University, New Haven, CT USA; 229 Yerevan Physics Institute, Yerevan, Armenia; 230 Centre de Calcul de l’Institut National de Physique Nucléaire et de Physique des Particules (IN2P3), Villeurbanne, France

## Abstract

A search for squarks and gluinos in final states containing hadronic jets, missing transverse momentum but no electrons or muons is presented. The data were recorded in 2015 by the ATLAS experiment in $\sqrt{s}=13~{\mathrm{TeV}}$ proton–proton collisions at the Large Hadron Collider. No excess above the Standard Model background expectation was observed in 3.2 $\mathrm{fb}^{-1}$ of analyzed data. Results are interpreted within simplified models that assume *R*-parity is conserved and the neutralino is the lightest supersymmetric particle. An exclusion limit at the 95 % confidence level on the mass of the gluino is set at 1.51 ${\mathrm{TeV}}$ for a simplified model incorporating only a gluino octet and the lightest neutralino, assuming the lightest neutralino is massless. For a simplified model involving the strong production of mass-degenerate first- and second-generation squarks, squark masses below 1.03 ${\mathrm{TeV}}$ are excluded for a massless lightest neutralino. These limits substantially extend the region of supersymmetric parameter space excluded by previous measurements with the ATLAS detector.

## Introduction

Supersymmetry (SUSY) [1–6] is a generalization of space-time symmetries that predicts new bosonic partners for the fermions and new fermionic partners for the bosons of the Standard Model (SM). If *R*-parity is conserved [7], SUSY particles (called sparticles) are produced in pairs and the lightest supersymmetric particle (LSP) is stable and represents a possible dark-matter candidate. The scalar partners of the left- and right-handed quarks, the squarks $\tilde{q}_{\mathrm {L}}$ and $\tilde{q}_{\mathrm {R}}$, mix to form two mass eigenstates $\tilde{q}_1$ and $\tilde{q}_2$ ordered by increasing mass. Superpartners of the charged and neutral electroweak and Higgs bosons also mix to produce charginos ($\tilde{\chi }^\pm$) and neutralinos ($\tilde{\chi }^0$). Squarks and the fermionic partners of the gluons, the gluinos ($\tilde{g}$), could be produced in strong-interaction processes at the Large Hadron Collider (LHC) [8] and decay via cascades ending with the stable LSP, which escapes the detector unseen, producing substantial missing transverse momentum ($\varvec{E}\mathrm {^{miss}_T}$).

The production of gluinos and squarks is the primary target for early supersymmetry searches in proton–proton (*pp*) collisions at a centre-of-mass energy of 13 ${\mathrm{TeV}}$ at the LHC because of the large expected cross-sections predicted for the production of supersymmetric particles which participate to the strong interaction. This document presents a search for these particles in final states containing only hadronic jets and large missing transverse momentum. Interest in this final state is motivated by the large number of *R*-parity-conserving models [9, 10] in which squarks (including anti-squarks) and gluinos can be produced in pairs ($\tilde{g} \tilde{g}$, $\tilde{q} \tilde{q}$, $\tilde{q} \tilde{g}$) and can decay through $\tilde{q} \rightarrow q \tilde{\chi }^0_1$ and $\tilde{g} \rightarrow q \bar{q} \tilde{\chi }^0_1$ to the lightest neutralino, $\tilde{\chi }^0_1$, assumed to be the LSP. Additional decay modes can include the production of charginos via $\tilde{q} \rightarrow q\tilde{\chi }^\pm$ (where $\tilde{q}$ and *q* are of different flavour) and $\tilde{g} \rightarrow q\bar{q}\tilde{\chi }^\pm$. Subsequent chargino decay to $W^{\pm }\tilde{\chi }^0_1$ can lead to still larger multiplicities of jets. The analysis presented here adopts the same analysis strategy as the previous ATLAS search designed for the analysis of the 7 ${\mathrm{TeV}}$ and 8 ${\mathrm{TeV}}$ data collected during Run 1 of the LHC, described in Refs. [11–15]. The CMS Collaboration has set limits on similar models in Refs. [16–21].

In this search, events with reconstructed electrons or muons are rejected to reduce the background from events with neutrinos ($W \rightarrow e\nu ,\mu \nu$) and to avoid any overlap with a complementary ATLAS search in final states with one lepton, jets and missing transverse momentum [22]. The selection criteria are optimized in the $(m_{\tilde{g}}, m_{\tilde{\chi }^0_1})$ and $(m_{\tilde{q}}, m_{\tilde{\chi }^0_1})$ planes, (where $m_{\tilde{g}}$, $m_{\tilde{q}}$ and $m_{\tilde{\chi }^0_1}$ are the gluino, squark and the LSP masses, respectively) for simplified models [23–25] in which all other supersymmetric particles are assigned masses beyond the reach of the LHC. Although interpreted in terms of SUSY models, the results of this analysis could also constrain any model of new physics that predicts the production of jets in association with missing transverse momentum.

## The ATLAS detector and data samples

The ATLAS detector [26] is a multi-purpose detector with a forward-backward symmetric cylindrical geometry and nearly 4$\pi$ coverage in solid angle.^1^ The inner tracking detector (ID) consists of pixel and silicon microstrip detectors covering the pseudorapidity region $|\eta |<2.5$, surrounded by a transition radiation tracker which improves electron identification over the region $|\eta |<2.0$. The innermost pixel layer, the insertable B-layer [27], was added between Run 1 and Run 2 of the LHC, at a radius of 33 mm around a new, narrower and thinner, beam pipe. The ID is surrounded by a thin superconducting solenoid providing an axial 2 T magnetic field and by a fine-granularity lead/liquid-argon (LAr) electromagnetic calorimeter covering $|\eta |<3.2$. A steel/scintillator-tile calorimeter provides hadronic coverage in the central pseudorapidity range ($|\eta |<1.7$). The endcap and forward regions ($1.5<|\eta |<4.9$) of the hadronic calorimeter are made of LAr active layers with either copper or tungsten as the absorber material. The muon spectrometer with an air-core toroid magnet system surrounds the calorimeters. Three layers of high-precision tracking chambers provide coverage in the range $|\eta |<2.7$, while dedicated chambers allow triggering in the region $|\eta |<2.4$.

The ATLAS trigger system [28] consists of two levels; the first level is a hardware-based system, while the second is a software-based system called the High-Level Trigger. The events used in this search were selected using a trigger logic that accepts events with a missing transverse momentum above 70 GeV, calculated using a sum over calorimeter cells. The trigger is 100 % efficient for the event selections considered in this analysis. Auxiliary data samples used to estimate the yields of background events were selected using triggers requiring at least one isolated electron ($p_{\text {T}} >24~{\mathrm{GeV}}$), muon ($p_{\text {T}} >20~{\mathrm{GeV}}$) or photon ($p_{\text {T}} >120~{\mathrm{GeV}}$). To increase the efficiency at high momenta, additional single-electron and single-muon triggers that do not require any isolation were included with thresholds of $p_{\text {T}} = 60~{\mathrm{GeV}}$ and $p_{\text {T}} = 50~{\mathrm{GeV}}$, respectively.

The dataset used in this analysis was collected in 2015 with the LHC colliding proton beams at a centre-of-mass energy of 13 ${\mathrm{TeV}}$, with 25 ns bunch spacing. The peak delivered instantaneous luminosity was $L = 5.2 \times 10^{33} ~\mathrm{cm^{-2} s^{-1}}$ and the mean number of additional *pp* interactions per bunch crossing in the dataset was $\left\langle \mu\right\rangle$ = 14. Application of beam, detector and data-quality criteria resulted in a total integrated luminosity of 3.2 $\mathrm{fb}^{-1}$. The uncertainty in the integrated luminosity is ±5 %. It is derived, following a methodology similar to that detailed in Ref. [29], from a preliminary calibration of the luminosity scale using a pair of *x*–*y* beam-separation scans performed in August 2015.

## Monte Carlo simulated samples

Simulated Monte Carlo (MC) data samples are used to optimize the selections, estimate backgrounds and assess the sensitivity to specific SUSY signal models.

**Fig. 1:**
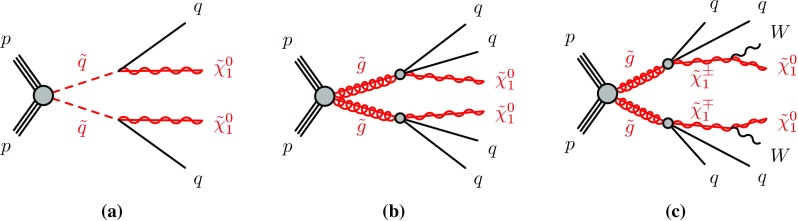
The decay topologies of **a** squark-pair production and **b**, **c** gluino-pair production, in the simplified models with direct decays of squarks and direct or one-step decays of gluinos

SUSY signals are described in this paper by simplified models. They are defined by an effective Lagrangian describing the interactions of a small number of new particles, typically assuming one production process and one decay channel with a 100 % branching fraction. Signal samples used to describe squark- and gluino-pair production, followed by the direct^2^ decays of squarks ($\tilde{q} \rightarrow q\tilde{\chi }^0_1$) and direct ($\tilde{g} \rightarrow q\bar{q} \tilde{\chi }^0_1$) or one-step^3^ ($\tilde{g} \rightarrow q\bar{q}'W \tilde{\chi }^0_1$) decays of gluinos as shown in Fig. 1, are generated with up to two extra partons in the matrix element using MG5_aMC@NLO event generator [30] interfaced to Pythia 8.186 [31]. The CKKW-L merging scheme [32] is applied with a scale parameter that is set to a quarter of the mass of the gluino for $\tilde{g} \tilde{g}$ production or of the squark for $\tilde{q} \tilde{q}$ production. The A14 [33] set of tuned parameters (tune) is used for underlying event together with the NNPDF2.3LO [34] parton distribution function (PDF) set. The EvtGen v1.2.0 program [35] is used to describe the properties of the *b*- and *c*- hadron decays in the signal samples and the background samples except those produced with Sherpa [36]. The signal cross-sections are calculated at next-to-leading order (NLO) in the strong coupling constant, adding the resummation of soft gluon emission at next-to-leading-logarithmic accuracy (NLO+NLL) [37–41]. The nominal cross-section is taken from an envelope of cross-section predictions using different PDF sets and factorization and renormalization scales, as described in Ref. [42], considering only light-flavour quarks (*u*, *d*, *s*, *c*). Cross-sections are evaluated assuming masses of 450 ${\mathrm{TeV}}$ for the light-flavour squarks in case of gluino- or gluinos in case of squark-pair production. The free parameters are $m_{\tilde{\chi }^0_1}$ and $m_{\tilde{q}}$ ($m_{\tilde{g}}$) for gluino-pair (squark-pair) production models.

A summary of the SM background processes together with the MC generators, cross-section calculation orders in $\alpha _\mathrm{s}$, PDFs, parton shower and tunes used is given in Table 1.

**Table 1:** The Standard Model background Monte Carlo simulation samples used in this paper. The generators, the order in $\alpha _\mathrm{s}$ of cross-section calculations used for yield normalization, PDF sets, parton showers and tunes used for the underlying event are shown

Physics process	Generator	Cross-section normalization	PDF set	Parton shower	Tune
$W(\rightarrow \ell \nu )$ + jets	Sherpa 2.1.1	NNLO	CT10	Sherpa	Sherpa default
$Z/\gamma ^{*}(\rightarrow \ell \bar{\ell })$ + jets	Sherpa 2.1.1	NNLO	CT10	Sherpa	Sherpa default
$\gamma$ + jets	Sherpa 2.1.1	LO	CT10	Sherpa	Sherpa default
$t\bar{t}$	Powheg-Box v2	NNLO+NNLL	CT10	Pythia 6.428	Perugia2012
Single top (*Wt*-channel)	Powheg-Box v2	NNLO+NNLL	CT10	Pythia 6.428	Perugia2012
Single top (*s*-channel)	Powheg-Box v2	NLO	CT10	Pythia 6.428	Perugia2012
Single top (*t*-channel)	Powheg-Box v1	NLO	CT10f4	Pythia 6.428	Perugia2012
$t\bar{t}+W/Z/WW$	MG5_aMC@NLO	NLO	NNPDF2.3LO	Pythia 8.186	A14
*WW*, *WZ*, *ZZ*	Sherpa 2.1.1	NLO	CT10	Sherpa	Sherpa default
Multi-jet	Pythia 8.186	LO	NNPDF2.3LO	Pythia 8.186	A14

The production of $\gamma$, *W* or *Z* bosons in association with jets [43] is simulated using the Sherpa 2.1.1 generator. For *W* or *Z* bosons, the matrix elements are calculated for up to two partons at NLO and up to two additional partons at leading order (LO) using the Comix [44] and OpenLoops [45] matrix-element generators, and merged with the Sherpa parton shower [46] using the ME+PS@NLO prescription [47]. Events containing a photon in association with jets are generated requiring a photon transverse momentum above 35 ${\mathrm{GeV}}$. For these events, matrix elements are calculated at LO with up to three or four partons depending on the $p_{\text {T}}$ of the photon, and merged with the Sherpa parton shower using the ME+PS@LO prescription [48]. In both cases (*W* / *Z*+jets or $\gamma$+jets production), the CT10 PDF set [49] is used in conjunction with dedicated parton shower-tuning developed by the authors of Sherpa. The *W* / *Z* + jets events are normalized to their NNLO cross-sections [50]. For the $\gamma$+jets process the LO cross-section, taken directly from the Sherpa MC generator, is multiplied by a correction factor as described in Sect. 7.

For the generation of $t\bar{t}$ and single-top processes in the *Wt* and *s*-channel [51] the Powheg-Box v2 [52] generator is used with the CT10 PDF set. The electroweak (EW) *t*-channel single-top events are generated using the Powheg-Box v1 generator. This generator uses the four-flavour scheme for the NLO matrix-element calculations together with the fixed four-flavour PDF set CT10f4 [49]. For this process, the decay of the top quark is simulated using MadSpin tool [53] preserving all spin correlations, while for all processes the parton shower, fragmentation, and the underlying event are generated using Pythia 6.428 [54] with the CTEQ6L1 [55] PDF set and the corresponding Perugia 2012 tune (P2012) [56]. The top quark mass is set to 172.5 ${\mathrm{GeV}}$. The $h_\mathrm{damp}$ parameter, which controls the $p_{\text {T}}$ of the first additional emission beyond the Born configuration, is set to the mass of the top quark. The main effect of this is to regulate the high-$p_{\text {T}}$ emission against which the ttbar system recoils [51]. The $t\bar{t}$ events are normalized to the NNLO+NNLL  [57, 58]. The *s*- and *t*-channel single-top events are normalized to the NLO cross-sections [59, 60], and the *Wt*-channel single-top events are normalized to the NNLO+NNLL [61, 62].

For the generation of $t\bar{t}$ + EW processes ($t\bar{t} + W/Z/WW$) [63], the MG5_aMC@NLO [30] generator at LO interfaced to the Pythia 8.186 parton-shower model is used, with up to two ($t\bar{t}+W$), one ($t\bar{t}+Z$) or no ($t\bar{t}+WW$) extra partons included in the matrix element. The ATLAS underlying-event tune A14 is used together with the NNPDF2.3LO PDF set. The events are normalized to their respective NLO cross-sections [64, 65].

Diboson processes (*WW*, *WZ*, *ZZ*) [66] are simulated using the Sherpa 2.1.1 generator. For processes with four charged leptons (4$\ell$), three charged leptons and a neutrino (3$\ell$+1$\nu$) or two charged leptons and two neutrinos (2$\ell$+2$\nu$), the matrix elements contain all diagrams with four electroweak vertices, and are calculated for up to one (4$\ell$, 2$\ell$+2$\nu$) or no partons (3$\ell$+1$\nu$) at NLO and up to three partons at LO using the Comix and OpenLoops matrix-element generators, and merged with the Sherpa parton shower using the ME+PS@NLO prescription. For processes in which one of the bosons decays hadronically and the other leptonically, matrix elements are calculated for up to one (*ZZ*) or no (*WW*, *WZ*) additional partons at NLO and for up to three additional partons at LO using the Comix and OpenLoops matrix-element generators, and merged with the Sherpa parton shower using the ME+PS@NLO prescription. In all cases, the CT10 PDF set is used in conjunction with a dedicated parton-shower tuning developed by the authors of Sherpa. The generator cross-sections are used in this case.

The multi-jet background is generated with Pythia 8.186 using the A14 underlying-event tune and the NNPDF2.3LO parton distribution functions.

For all Standard Model background samples the response of the detector to particles is modelled with a full ATLAS detector simulation [67] based on Geant4 [68]. Signal samples are prepared using a fast simulation based on a parameterization of the performance of the ATLAS electromagnetic and hadronic calorimeters [69] and on Geant4 elsewhere.

All simulated events are overlaid with multiple *pp* collisions simulated with the soft QCD processes of Pythia 8.186 using the A2 tune [33] and the MSTW2008LO parton distribution functions [70]. The simulations are not reweighted to match the distribution of the mean number of interactions observed in data. It was checked that the effect of such pile-up reweighting is completely negligible.

## Object reconstruction and identification

The reconstructed primary vertex of the event is required to be consistent with the luminous region and to have at least two associated tracks with $p_{\text {T}} > 400$ ${\mathrm{MeV}}$. When more than one such vertex is found, the vertex with the largest $\sum p_{\text {T}} ^2$ of the associated tracks is chosen.

Jet candidates are reconstructed using the anti-$k_{t}$ jet clustering algorithm [71, 72] with jet radius parameter of 0.4 and starting from clusters of calorimeter cells [73]. The jets are corrected for energy from pile-up using the method suggested in Ref. [74]: a contribution equal to the product of the jet area and the median energy density of the event is subtracted from the jet energy [75]. Further corrections, referred to as the jet energy scale corrections, are derived from MC simulation and data and used to calibrate on average the energies of jets to the scale of their constituent particles [76]. Only jet candidates with $p_\mathrm {T}> 20~{\mathrm{GeV}}$ and $|\eta |<2.8$ after all corrections are retained. An algorithm based on boosted decision trees, ‘MV2c20’ [77], is used to identify jets containing a *b*-hadron (*b*-jets), with an operating point corresponding to an efficiency of 77 % in simulated $t\bar{t}$ events, along with a rejection factor of 140 for gluon and light-quark jets and of 4.5 for charm jets [77, 78]. Candidate *b*-tagged jets are required to have $p_\mathrm {T}> 50~{\mathrm{GeV}}$ and $|\eta |<2.5$. Events with jets originating from detector noise and non-collision background are rejected if the jets fail to satisfy the ‘LooseBad’ quality criteria, or if at least one of the two leading jets with $p_{\text {T}} >100~{\mathrm{GeV}}$ fails to satisfy the ‘TightBad’ quality criteria, both described in Ref. [79]. These selections affect less than 1 % of the events used in the search.

Two different classes of reconstructed lepton candidates (electrons or muons) are used in this analysis. When selecting samples used for the search, events containing a ‘baseline’ electron or muon are rejected. The selections applied to identify baseline leptons are designed to maximize the efficiency with which *W*+jets and top quark background events are rejected. When selecting ‘control region’ samples for the purpose of estimating residual *W*+jets and top quark backgrounds, additional requirements are applied to leptons to ensure greater purity of the these backgrounds. These leptons are referred to as ‘high-purity’ leptons below and form a subset of the baseline leptons.

Baseline muon candidates are formed by combining information from the muon spectrometer and inner tracking detectors as described in Ref. [80] and are required to have $p_\mathrm {T}> 10~ {\mathrm{GeV}}$ and $|\eta | <2.7$. High-purity muon candidates must additionally have $|\eta |<2.4$, the significance of the transverse impact parameter with respect to the primary vertex, $|d_0^{\mathrm {PV}}|/\sigma (d_0^{\mathrm {PV}})<$ 3, the longitudinal impact parameter with respect to the primary vertex $|z_0^{\mathrm {PV}} \mathrm {sin}(\theta )|<$ 0.5 mm, and to satisfy ‘GradientLoose’ isolation requirements described in Ref. [80] which rely on the use of tracking-based and calorimeter-based variables and implement a set of $\eta$- and $p_{\text {T}}$-dependent criteria. The leading muon is also required to have $p_{\text {T}} > 25~{\mathrm{GeV}}$.

Baseline electron candidates are reconstructed from an isolated electromagnetic calorimeter energy deposit matched to an ID track and are required to have $p_\mathrm {T}> 10~{\mathrm{GeV}}$, $|\eta | < 2.47$, and to satisfy ‘Loose’ likelihood-based identification criteria described in Ref. [81]. High-purity electron candidates additionally must satisfy ‘Tight’ selection criteria described in Ref. [81], and the leading electron must have $p_{\text {T}} >25~{\mathrm{GeV}}$. They are also required to have $|d_0^{\mathrm {PV}}|/\sigma (d_0^{\mathrm {PV}})<$ 5, $|z_0^{\mathrm {PV}} \mathrm {sin}(\theta )|<$ 0.5 mm, and to satisfy similar isolation requirements as those applied to high-purity muons.

After the selections described above, ambiguities between candidate jets with $|\eta |<2.8$ and leptons are resolved as follows: first, any such jet candidate lying within a distance $\Delta R\equiv \sqrt{(\Delta y)^2+(\Delta \phi )^2}=0.2$ of a baseline electron is discarded; then any baseline lepton candidate remaining within a distance $\Delta R =0.4$ of any surviving jet candidate is discarded, except in the case where the lepton is a muon (which can radiate a photon and be misidentified as a jet) and the number of tracks associated with the jet is less than three.

Additional ambiguities between electrons and muons in a jet, originating from the decays of hadrons, are resolved to avoid double counting and/or remove non-isolated leptons: the electron is discarded if a baseline electron and a baseline muon share the same ID track. If two baseline electrons are within $\Delta R$ = 0.05, the electron with the lowest $p_{\text {T}}$ is discarded.

The measurement of the missing transverse momentum vector $\varvec{E}\mathrm {^{miss}_T}$ (and its magnitude $E_{\text {T}}^{\text {miss}}$) is based on the calibrated transverse momenta of all electron, muon, photon and jet candidates and all tracks originating from the primary vertex and not associated with such objects [82].

Reconstructed photons, although not used in the main signal-event selection, are selected in the region used to constrain the *Z*+jets background, as explained in Sect. 7. Photon candidates are required to satisfy $p_\mathrm {T}> 130~{\mathrm{GeV}}$ and $|\eta | < 2.37$, to satisfy photon shower shape and electron rejection criteria [83], and to be isolated. Ambiguities between candidate jets and photons (when used in the event selection) are resolved by discarding any jet candidates lying within $\Delta R$ = 0.4 of a photon candidate. Additional selections to remove ambiguities between electrons or muons and photons are applied such that the photon is discarded if it is within $\Delta R$ = 0.4 of an electron or muon.

Corrections derived from data control samples are applied to account for differences between data and simulation for the lepton trigger and reconstruction efficiencies, the lepton momentum/energy scale and resolution, and for the efficiency and mis-tag rate of the *b*-tagging algorithm.

## Analysis strategy and fit description

To search for a possible signal, selections are defined to enhance the signal relative to the SM background. These signal region (SR) selections are optimized to maximize the expected significance for each model considered using MC simulation for the signal and the SM backgrounds. To estimate the SM backgrounds in a consistent and robust fashion, corresponding control regions (CRs) are defined for each of the signal regions. They are chosen to be non-overlapping with the SR selections in order to provide independent data samples enriched in particular background sources, and are used to normalize the background MC simulation. The CR selections are optimized to have negligible SUSY signal contamination for the models near the previously excluded boundary [14], while minimizing the systematic uncertainties arising from the extrapolation of the CR event yields to estimate backgrounds in the SR. Cross-checks of the background estimates are performed with data in several validation regions (VRs) selected with requirements such that these regions do not overlap with the CR and SR selections, again with a low expected signal contamination.

To extract the final results, three different classes of likelihood fit are employed: background-only, model-independent and model-dependent fits [84]. A background-only fit is used to estimate the background yields in each SR. The fit is performed using as constraints only the observed event yields from the CRs associated with the SR, but not the SR itself. It is assumed that signal events from physics beyond the Standard Model (BSM) do not contribute to these yields. The scale factors ($\mu _{W\mathrm +jets}$, $\mu _{Z\mathrm +jets}$, $\mu _\mathrm{Top}$, $\mu _{\text {Multi-jet}}$) are fitted in each CR attached to a SR. The expected background in the SR is based on the yields predicted by simulation, corrected by the scale factors derived from the fit. The systematic uncertainties and the MC statistical uncertainties in the expected values are included in the fit as nuisance parameters which are constrained by Gaussian distributions with widths corresponding to the sizes of the uncertainties considered and by Poisson distributions, respectively. The background-only fit is also used to estimate the background event yields in the VRs.

If no excess is observed, a model-independent fit is used to set upper limits on the number of BSM signal events in each SR. This fit proceeds in the same way as the background-only fit, except that the number of events observed in the SR is added as an input to the fit, and the BSM signal strength, constrained to be non-negative, is added as a free parameter. The observed and expected upper limits at 95 % confidence level (CL) on the number of events from BSM phenomena for each signal region ($S_\mathrm{obs}^{95}$ and $S_\mathrm{exp}^{95}$) are derived using the $CL_\mathrm{s}$ prescription [85], neglecting any possible signal contamination in the control regions. These limits, when normalized by the integrated luminosity of the data sample, may be interpreted as upper limits on the visible cross-section of BSM physics ($\langle \epsilon \sigma \rangle _\mathrm{obs}^{95}$), where the visible cross-section is defined as the product of production cross-section, acceptance and efficiency. The model-independent fit is also used to compute the one-sided *p*-value ($p_0$) of the background-only hypothesis, which quantifies the statistical significance of an excess.

Finally, model-dependent fits are used to set exclusion limits on the signal cross-sections for specific SUSY models. Such a fit proceeds in the same way as the model-independent fit, except that both the yield in the signal region and the signal contamination in the CRs are taken into account. Correlations between signal and background systematic uncertainties are taken into account where appropriate. Signal-yield systematic uncertainties due to detector effects and the theoretical uncertainties in the signal acceptance are included in the fit.

## Event selection and signal regions definitions

Due to the high mass scale expected for the SUSY models considered in this study, the ‘effective mass’, $m_{\mathrm {eff}}$, is a powerful discriminant between the signal and most SM backgrounds. When selecting events with at least $N_\mathrm{j}$ jets, $m_{\mathrm {eff}}(N_\mathrm{j})$ is defined to be the scalar sum of the transverse momenta of the leading $N_\mathrm{j}$ jets and $E_{\text {T}}^{\text {miss}}$ . Requirements placed on $m_{\mathrm {eff}}$ and $E_{\text {T}}^{\text {miss}}$ form the basis of this search by strongly suppressing the multi-jet background where jet energy mismeasurement generates missing transverse momentum. The final signal selection uses requirements on both $m_{\mathrm {eff}}(\mathrm{incl.})$, which sums over all jets with $p_\mathrm {T}>50~{\mathrm{GeV}}$ and $E_{\text {T}}^{\text {miss}}$ , which is required to be larger than 200 ${\mathrm{GeV}}$.

Following the object reconstruction described in Sect. 4, events are discarded if a baseline electron or muon with $p_{\text {T}} >10~{\mathrm{GeV}}$ remains, or if they contain a jet failing to satisfy quality selection criteria designed to suppress detector noise and non-collision backgrounds (described in Sect. 4). Events are also rejected if no jets with $p_{\text {T}} >50~{\mathrm{GeV}}$ are found. Reconstructed photons and hadronically decaying $\tau$-leptons are not used in SR selections.

In order to maximize the sensitivity in the $(m_{\tilde{g}},m_{\tilde{q}})$ plane, a variety of signal regions are defined. Squarks typically generate at least one jet in their decays, for instance through $\tilde{q} \rightarrow q \tilde{\chi }^0_1$, while gluinos typically generate at least two jets, for instance through $\tilde{g} \rightarrow q \bar{q} \tilde{\chi }^0_1$. Processes contributing to $\tilde{q} \tilde{q}$ and $\tilde{g} \tilde{g}$ final states therefore lead to events containing at least two or four jets, respectively. Decays of heavy SUSY and SM particles produced in longer $\tilde{q}$ and $\tilde{g}$ decay cascades (e.g. $\tilde{\chi }^\pm _1 \rightarrow qq'\tilde{\chi }^0_1$) tend to further increase the jet multiplicity in the final state.

Seven inclusive SRs characterized by increasing minimum jet multiplicity from two to six, are defined in Table 2. Some of them require the same jet-multiplicity, but are distinguished by increasing background rejection, ranging from ‘loose’ (labelled ‘l’) to ‘tight’ (labelled ‘t’).

In each region, different thresholds are applied on jet momenta and on $\Delta \phi (\text {jet},\varvec{E}\mathrm {^{miss}_T})_\mathrm {min}$, which is defined to be the smallest azimuthal separation between $\varvec{E}\mathrm {^{miss}_T}$ and the momenta of any of the reconstructed jets with $p_\mathrm {T}>50~{\mathrm{GeV}}$. Requirements on $\Delta \phi (\text {jet},\varvec{E}\mathrm {^{miss}_T})_\mathrm {min}$ and $E_{\text {T}}^{\text {miss}}/m_{\mathrm {eff}}(N_\mathrm{j})$ are designed to reduce the background from multi-jet processes. For the SRs which are optimized for squark-pair (gluino-pair) production followed by the direct decay of squarks (gluinos), the selection requires $\Delta \phi (\text {jet},\varvec{E}\mathrm {^{miss}_T})_\mathrm {min}>0.8$ ($\Delta \phi (\text {jet},\varvec{E}\mathrm {^{miss}_T})_\mathrm {min}>0.4$) using up to three leading jets present in the event. For the SRs requiring at least four jets in the final state, an additional requirement $\Delta \phi (\text {jet},\varvec{E}\mathrm {^{miss}_T})_\mathrm {min}>0.2$ is placed on all jets. Signal region 2jm makes use of the presence of jets due to initial-state radiation by requiring a higher $p_{\text {T}}$ threshold for the most energetic jet in the event, and is optimized to target models with small mass differences between the SUSY particles (compressed scenarios).

In the 2-jet SRs the requirement on $E_{\text {T}}^{\text {miss}}/m_{\mathrm {eff}}(N_\mathrm{j})$ is replaced by a requirement on $E_{\text {T}}^{\text {miss}}/\sqrt{H_\mathrm{T}}$ (where $H_\mathrm{T}$ is defined as the scalar sum of the transverse momenta of all jets), which was found to lead to enhanced sensitivity to models characterized by $\tilde{q} \tilde{q}$ production. In the other regions, additional suppression of background processes is based on the aplanarity variable, which is defined as $A = 3/2 \lambda _3$, where $\lambda _3$ is the smallest eigenvalue of the normalized momentum tensor of the jets [86].

**Table 2:**
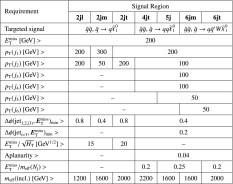
Selection criteria and targeted signal model used to define each of the signal regions in the analysis. Each SR is labelled with the inclusive jet multiplicity considered (‘2j’, ‘4j’ etc.) together with the degree of background rejection. The latter is denoted by labels ‘l’ (‘loose’), ‘m’ (‘medium’) and ‘t’ (‘tight’). The $E_{\text {T}}^{\text {miss}}/m_{\mathrm {eff}}(N_\mathrm{j})$ cut in any $N_\mathrm{j}$-jet channel uses a value of $m_{\mathrm {eff}}$ constructed from only the leading $N_\mathrm{j}$ jets ($m_{\mathrm {eff}}(N_\mathrm{j})$). However, the final $m_{\mathrm {eff}}(\mathrm{incl.})$ selection, which is used to define the signal regions, includes all jets with $p_{\text {T}} >50~{\mathrm{GeV}}$

## Background estimation and validation

Standard Model background processes contribute to the event counts in the signal regions. The dominant sources are: $Z+$jets, $W+$jets, top quark pairs, single top quarks, dibosons and multi-jet production. Diboson production is estimated with MC simulated data normalized to NLO cross-section predictions, as described in Sect. 3. Most of the *W*+jets background is composed of $W\rightarrow \tau \nu$ events in which the $\tau$-lepton decays to hadrons, with additional contributions from $W\rightarrow e\nu , \mu \nu$ events in which no baseline electron or muon is reconstructed. The largest part of the *Z*+jets background comes from the irreducible component in which $Z\rightarrow \nu \bar{\nu }$ decays generate large $E_{\text {T}}^{\text {miss}}$. Top quark pair production followed by semileptonic decays, in particular $t \bar{t} \rightarrow b \bar{b} \tau \nu q q'$ (with the $\tau$-lepton decaying to hadrons), as well as single-top-quark events, can also generate large $E_{\text {T}}^{\text {miss}}$ and satisfy the jet and lepton-veto requirements. The multi-jet background in the signal regions is due to missing transverse momentum from misreconstruction of jet energies in the calorimeters, as well as neutrino production in semileptonic decays of heavy-flavour hadrons. After applying the requirements based on $\Delta \phi (\text {jet},\varvec{E}\mathrm {^{miss}_T})_\mathrm {min}$ and $E_{\text {T}}^{\text {miss}}/m_{\mathrm {eff}}(N_\mathrm{j})$ listed in Table 2 the remaining multi-jet background is negligible.

**Table 3:** Control regions used in the analysis. Also listed are the main targeted background in the SR in each case, the process used to model the background, and the main CR requirement(s) used to select this process. The transverse momenta of high-purity leptons (photons) used to select CR events must exceed 25 (130) ${\mathrm{GeV}}$. The jet $p_{\text {T}}$ thresholds and $m_{\mathrm {eff}}(\mathrm{incl.})$ selections match those used in the corresponding SRs

CR	SR background	CR process	CR selection
CR$\gamma$	$Z(\rightarrow \nu \bar{\nu })$+jets	$\gamma$+jets	Isolated photon
CRQ	Multi-jet	Multi-jet	SR with reversed requirements on (i) $\Delta \phi (\text {jet},\varvec{E}\mathrm {^{miss}_T})_\mathrm {min}$
			and (ii) $E_{\text {T}}^{\text {miss}}/m_{\mathrm {eff}}(N_\mathrm{j})$ or $E_{\text {T}}^{\text {miss}}/\sqrt{H_\mathrm{T}}$
CRW	$W(\rightarrow \ell \nu )$+jets	$W(\rightarrow \ell \nu )$+jets	30 ${\mathrm{GeV}}$ $<m_\mathrm{T}(\ell ,E_{\text {T}}^{\text {miss}}) < 100$ ${\mathrm{GeV}}$, *b*-veto
CRT	$t\bar{t}$(+EW) and single top	$t\bar{t}\rightarrow b\bar{b}qq'\ell \nu$	30 ${\mathrm{GeV}}$ $<m_\mathrm{T}(\ell ,E_{\text {T}}^{\text {miss}}) < 100$ ${\mathrm{GeV}}$, *b*-tag

In order to estimate the backgrounds in a consistent and robust fashion, four control regions are defined for each of the seven signal regions, giving 28 CRs in total. The CR selections are optimized to maintain adequate statistical precision while minimizing the systematic uncertainties arising from the extrapolation of the CR event yield to estimate the background in the SR. This latter requirement is addressed through the use of CR jet $p_{\text {T}}$ thresholds and $m_{\mathrm {eff}}$(incl.) selections which match those used in the SR. The CR definitions are listed in Table 3.

The CR$\gamma$ region is used to estimate the contribution of $Z(\rightarrow \nu \bar{\nu })$+jets background events to each SR by selecting a sample of $\gamma$+jets events with $p_{\text {T}} (\gamma )>130~{\mathrm{GeV}}$ and then treating the reconstructed photon as contributing to $E_{\text {T}}^{\text {miss}}$. For $p_{\text {T}} (\gamma )$ significantly larger than $m_Z$ the kinematic properties of such events strongly resemble those of *Z*+jets events [13]. In order to reduce the theoretical uncertainties associated with the $Z/\gamma ^*$+jets background expectations in SRs arising from the use of LO $\gamma$+jets cross-sections, a correction factor is applied to the CR$\gamma$ events. This correction factor, $\kappa =1.5 \pm 0.1$, is determined by comparing CR$\gamma$ observations with those in a highly populated auxiliary control region defined by selecting events with two electrons or muons for which the invariant mass lies within 25 ${\mathrm{GeV}}$ of the mass of the *Z* boson, satisfying $200~{\mathrm{GeV}}< | \varvec{E}\mathrm {^{miss}_T} + \varvec{p}\mathrm {_T}(\ell \bar{\ell }) | < 300~{\mathrm{GeV}}$, together with at least two jets.

The CRW and CRT regions aim to select samples rich in $W(\rightarrow \ell \nu )$+jets and semileptonic $t\bar{t}$ background events respectively. Consequently, they differ in their number of *b*-jets (zero or greater or equal to one respectively) but apply the same selection requirements on the transverse mass $m_\mathrm{T}$ formed by the $E_\mathrm{T}^{\mathrm {miss}}$ and a high-purity lepton with $p_\mathrm{T}$ > 25 ${\mathrm{GeV}}$. These samples are used to estimate respectively the *W*+jets and combined $t\bar{t}$ and single-top background populations, treating the lepton as a jet with the same momentum to model background events in which a hadronically decaying $\tau$-lepton is produced or events in which no baseline electron or muon is reconstructed because it is outside the detector acceptance or below the required $p_{\text {T}}$ threshold. The CRW and CRT selections omit the SR selection requirements on $\Delta \phi (\text {jet},\varvec{E}\mathrm {^{miss}_T})_\mathrm {min}$ or $E_{\text {T}}^{\text {miss}}/m_{\mathrm {eff}}(N_\mathrm{j})$ ($E_{\text {T}}^{\text {miss}}/\sqrt{H_\mathrm{T}}$ where appropriate) in order to increase the number of CR data events without significantly increasing the theoretical uncertainties associated with the background estimation procedure.

The CRQ region uses reversed selection requirements on $\Delta \phi (\text {jet},\varvec{E}\mathrm {^{miss}_T})_\mathrm {min}$ and on $E_{\text {T}}^{\text {miss}}/m_{\mathrm {eff}}(N_\mathrm{j})$ (or $E_{\text {T}}^{\text {miss}}/\sqrt{H_\mathrm{T}}$ where appropriate) to produce samples enriched in multi-jet background events.

**Fig. 2:**
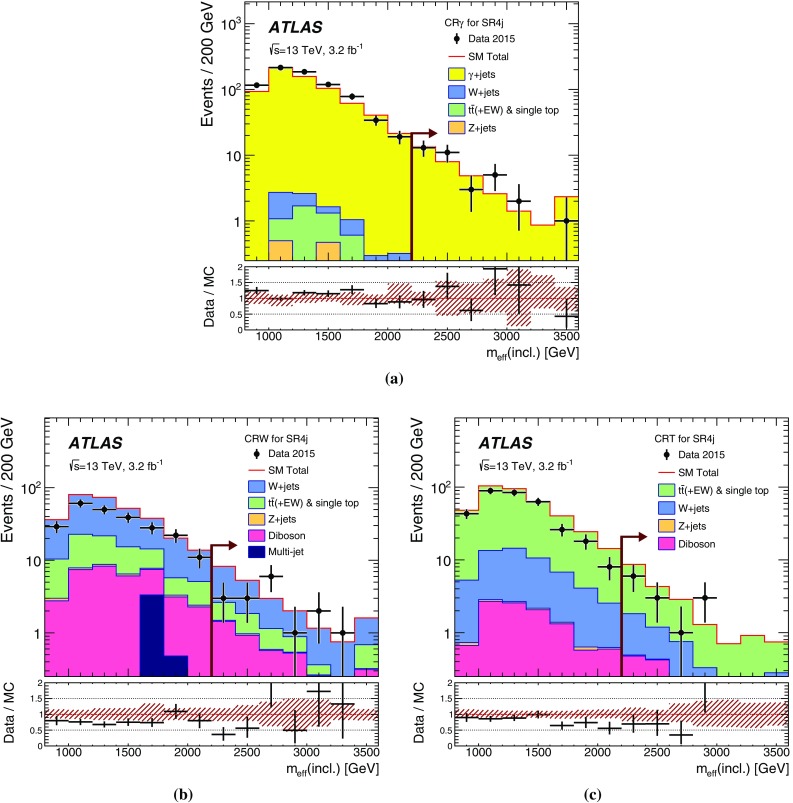
Observed $m_{\mathrm {eff}}(\mathrm{incl.})$ distributions in control regions **a** CR$\gamma$, **b** CRW and **c** CRT after selecting events with $E_{\text {T}}^{\text {miss}} > 200 ~{\mathrm{GeV}}$ and at least four energetic jets with the corresponding transverse momenta as indicated in Table 2 for SR 4jt. No selection requirements on $\Delta \phi (\text {jet},\varvec{E}\mathrm {^{miss}_T})_\mathrm {min}$ or $E_{\text {T}}^{\text {miss}}/m_{\mathrm {eff}}(N_\mathrm{j})$ are applied in these distributions. The arrows indicate the values at which the requirements on $m_{\mathrm {eff}}(\mathrm{incl.})$ are applied. The *histograms* denote the pre-fit MC background expectations, normalized to cross-section times integrated luminosity. The last bin includes the overflow. In the *lower panels* the hatched (*red*) error bands denote the combined experimental, MC statistical and theoretical modelling uncertainties

As an example, the $m_{\mathrm {eff}}(\mathrm{incl.})$ distributions in control regions associated with SR 4jt are shown in Fig. 2. In all CRs, the data are consistent with the pre-fit MC background prediction within uncertainties, although the overall normalization is lower by approximately one standard deviation.

The background estimation procedure is validated by comparing the numbers of events observed in the VRs to the corresponding SM background expectations obtained from the background-only fits. Several VR samples are selected with requirements distinct from those used in the CRs, which maintain a low probability of signal contamination.

The CR$\gamma$ estimates of the $Z(\rightarrow \nu \bar{\nu })$+jets background are validated using the samples of $Z(\rightarrow \ell \bar{\ell })$+jets events selected by requiring high-purity lepton pairs of opposite sign and identical flavour for which the dilepton invariant mass lies within 25 ${\mathrm{GeV}}$ of the mass of the *Z* boson (VRZ). In VRZ, the leptons are treated as contributing to $E_{\text {T}}^{\text {miss}}$.

The CRW and CRT estimates of the *W*+jets and top quark background are validated with the same CRW and CRT selections, but reinstating the requirement on $\Delta \phi (\text {jet},\varvec{E}\mathrm {^{miss}_T})_\mathrm {min}$ and $E_{\text {T}}^{\text {miss}}/m_{\mathrm {eff}}(N_\mathrm{j})$ (or $E_{\text {T}}^{\text {miss}}/\sqrt{H_\mathrm{T}}$ as appropriate), and treating the lepton either as a jet (VRW, VRT) or as contributing to $E_{\text {T}}^{\text {miss}}$ (VRW$\nu$, VRT$\nu$).

The CRQ estimates of the multi-jet background are validated with VRs for which the CRQ selection is applied, but with the SR $E_{\text {T}}^{\text {miss}}/m_{\mathrm {eff}}(N_\mathrm{j})$ ($E_{\text {T}}^{\text {miss}}/\sqrt{H_\mathrm{T}}$) requirement reinstated (VRQa), or with a requirement of an intermediate value of $\Delta \phi (\text {jet},\varvec{E}\mathrm {^{miss}_T})_\mathrm {min}$ applied (VRQb).

**Fig. 3:**
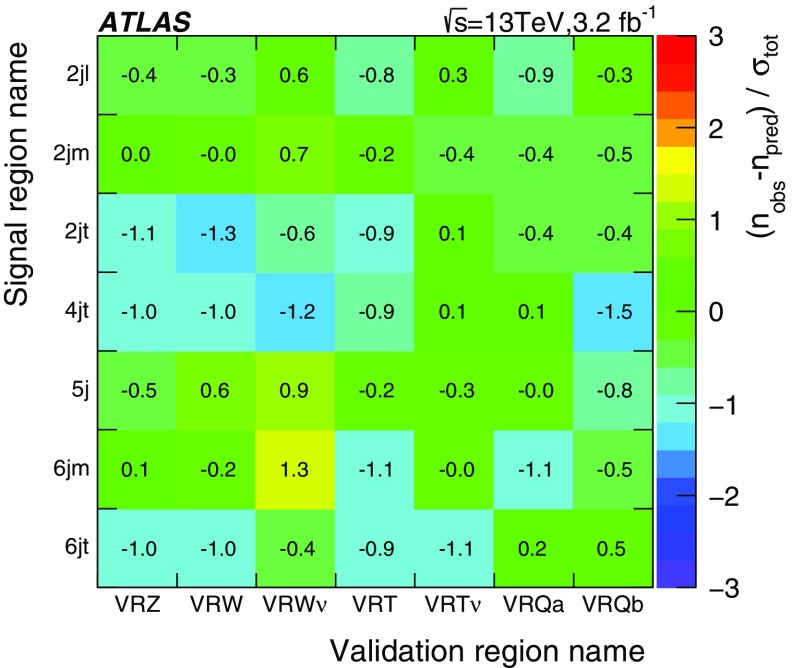
Differences between the numbers of observed events in data and the SM background predictions for each VR, expressed as a fraction of the total uncertainty which combines the uncertainty on the background expectations, and the expected statistical uncertainty of the test obtained from the number of expected events

The results of the validation procedure are shown in Fig. 3. The entries in the matrix are the differences between the numbers of observed and expected events expressed as fractions of the one-standard deviation $(1\sigma )$ uncertainties on the latter. Most VR observations lie within $1\sigma$ of the background expectations, with the largest discrepancy out of 49 VRs being $-1.5\sigma$ the CRQb associated with the SR 4jt.

## Systematic uncertainties

Systematic uncertainties in background estimates arise from the use of extrapolation factors which relate observations in the control regions to background expectations in the signal regions, and from the MC modelling of minor backgrounds.

The overall background uncertainties, detailed in Table 4, range from 8 % in SR 2jl to 29 % in SR 6jt. In SR 2jl the loose selection minimizes theoretical uncertainties and the impact of statistical fluctuations in the CRs, while the opposite is true in SR 6jt.

For the backgrounds estimated with MC simulation-derived extrapolation factors, the primary common sources of systematic uncertainty are the jet energy scale (JES) calibration, jet energy resolution (JER), theoretical uncertainties, and limited event yields in the MC samples and data CRs. Correlations between uncertainties (for instance between JES or JER uncertainties in CRs and SRs) are taken into account where appropriate.

The JES uncertainty was measured using the techniques described in Refs. [76, 87, 88]. The JER uncertainty is estimated using the methods discussed in Refs. [76, 89]. An additional uncertainty in the modelling of energy not associated with reconstructed objects, used in the calculation of $E_{\text {T}}^{\text {miss}}$ and measured with unassociated charged tracks, is also included. The combined JES, JER and $E_{\text {T}}^{\text {miss}}$ (Jet/$E_{\text {T}}^{\text {miss}}$) uncertainty ranges from <1 % of the expected background in 2-jet SRs to 5 % in SR 6jt.

Uncertainties arising from theoretical modelling of background processes are evaluated by comparing samples produced with different MC generators. The *W* / *Z*+jets events generated with Sherpa are compared to events generated with MG5_aMC@NLO at leading order and interfaced to the Pythia 8.186 parton shower model. Uncertainties in the modelling of top quark pair production are estimated by comparing Powheg-Box to aMc@Nlo [90], and by accounting for different generator and radiation tunes. Uncertainties associated with PDF modelling of top quark pair production are found to be negligible. Uncertainties in diboson production due to PDF, renormalization, factorization and resummation scale uncertainties (estimated by increasing and decreasing the scales used in the MC generators by a factor of two) are accounted for by applying a uniform 50 % uncertainty in all SRs, and are the dominant source of uncertainty in SRs 2jl and 2jm. Uncertainties associated with the modelling of *Z*+jets production are largest in the SRs with tight selection cuts (up to 14 %). The statistical uncertainty arising from the use of MC samples is largest (8 %) in SR 6jt. The uncertainties arising from the data-driven correction procedure applied to events selected in the CR$\gamma$ region, described in Sect. 7, are included in Table 4 under ‘CR$\gamma$ corr. factor’ and reach a value of 4 % in most of the SRs. The impact of lepton reconstruction uncertainties, and of the uncertainties related to the *b*-tag/*b*-veto efficiency, on the overall background uncertainty are found to be negligible for all SRs. The total background uncertainties for all SRs, broken down into the main contributing sources, are summarized in Table 4.

**Table 4:** Breakdown of the dominant systematic uncertainties in the background estimates. The individual uncertainties can be correlated, and do not necessarily add in quadrature to the total background uncertainty. $\Delta \mu$ uncertainties are the result of the control region statistical uncertainties and the systematic uncertainties entering a specific control region. In brackets, uncertainties are given relative to the expected total background yield, also presented in the Table. Empty cells (indicated by a ‘–’) correspond to uncertainties lower than 1 per mil

Channel	2jl	2jm	2jt	4jt	5j	6jm	6jt
Total bkg	283	191	23	4.6	13.2	6.9	4.2
Total bkg unc.	$\pm 24$ [$8~\%$]	$\pm 21$ [$11~\%$]	$\pm 4$ [$17~\%$]	$\pm 1.1$ [$24~\%$]	$\pm 2.2$ [$17~\%$]	$\pm 1.5$ [$22~\%$]	$\pm 1.2$ [$29~\%$]
MC statistics	–	$\pm 2.3$ [$1~\%$]	$\pm 0.5$ [$2~\%$]	$\pm 0.31$ [$7~\%$]	$\pm 0.5$ [$4~\%$]	$\pm 0.4$ [$6~\%$]	$\pm 0.32$ [$8~\%$]
$\Delta \mu _{Z\mathrm +jets}$	$\pm 7$ [$2~\%$]	$\pm 6$ [$3~\%$]	$\pm 2.5$ [$11~\%$]	$\pm 0.7$ [$15~\%$]	$\pm 1.0$ [$8~\%$]	$\pm 0.8$ [$12~\%$]	$\pm 0.7$ [$17~\%$]
$\Delta \mu _{W\mathrm +jets}$	$\pm 10$ [$4~\%$]	$\pm 8$ [$4~\%$]	$\pm 1.2$ [$5~\%$]	$\pm 0.5$ [$11~\%$]	$\pm 1.1$ [$8~\%$]	$\pm 0.7$ [$10~\%$]	$\pm 0.5$ [$12~\%$]
$\Delta \mu$ Top	$\pm 1.8$ [$1~\%$]	$\pm 2.0$ [$1~\%$]	$\pm 0.23$ [$1~\%$]	$\pm 0.26$ [$6~\%$]	$\pm 0.4$ [$3~\%$]	$\pm 0.24$ [$3~\%$]	$\pm 0.22$ [$5~\%$]
$\Delta \mu _{\text {Multi-jet}}$	$\pm 0.05$ [$0~\%$]	$\pm 0.09$ [$0~\%$]	$\pm 0.1$ [$0~\%$]	–	–	–	–
CR$\gamma$ corr. factor	$\pm 11$ [$4~\%$]	$\pm 7$ [$4~\%$]	$\pm 1.0$ [$4~\%$]	$\pm 0.17$ [$4~\%$]	$\pm 0.4$ [$3~\%$]	$\pm 0.21$ [$3~\%$]	$\pm 0.15$ [$4~\%$]
Theory *Z*	$\pm 8$ [$3~\%$]	$\pm 4$ [$2~\%$]	$\pm 2.4$ [$10~\%$]	$\pm 0.6$ [$13~\%$]	$\pm 0.6$ [$5~\%$]	$\pm 0.5$ [$7~\%$]	$\pm 0.6$ [$14~\%$]
Theory *W*	$\pm 2.9$ [$1~\%$]	$\pm 2.5$ [$1~\%$]	$\pm 0.5$ [$2~\%$]	$\pm 0.29$ [$6~\%$]	$\pm 0.7$ [$5~\%$]	$\pm 0.5$ [$7~\%$]	$\pm 0.4$ [$10~\%$]
Theory top	$\pm 2.1$ [$1~\%$]	$\pm 2.1$ [$1~\%$]	$\pm 0.28$ [$1~\%$]	$\pm 0.12$ [$3~\%$]	$\pm 0.8$ [$6~\%$]	$\pm 0.4$ [$6~\%$]	$\pm 0.13$ [$3~\%$]
Theory diboson	$\pm 15$ [$5~\%$]	$\pm 15$ [$8~\%$]	$\pm 1.0$ [$4~\%$]	–	$\pm 1.0$ [$8~\%$]	–	–
Jet/$E_{\text {T}}^{\text {miss}}$	$\pm 0.7$ [$0~\%$]	$\pm 0.6$ [$0~\%$]	$\pm 0.09$ [$0~\%$]	$\pm 0.1$ [$2~\%$]	$\pm 0.4$ [$3~\%$]	$\pm 0.21$ [$3~\%$]	$\pm 0.19$ [$5~\%$]

## Results, interpretation and limits

**Fig. 4:**
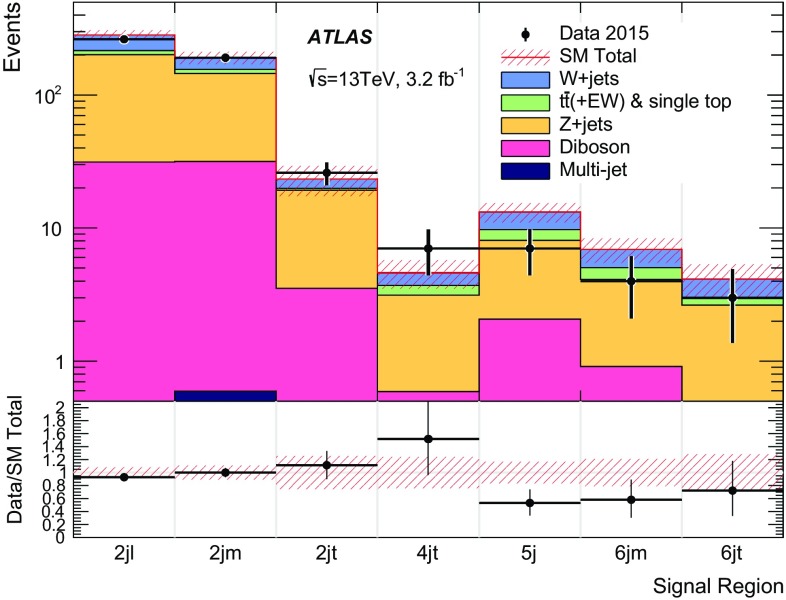
Comparison of the observed and expected event yields as a function of signal region. The background expectations are those obtained from the background-only fits, presented in Table 5

The number of events observed in the data and the number of SM events expected to enter each of the signal regions, determined using the background-only fit, are shown in Table 5 and Fig. 4. The pre-fit background expectations are also shown in Table 5 for comparison. The normalisation factors extracted simultaneously through the fit range for the different signal regions between 0.7 and 1.2 for *W*+jets, 0.4 and 0.8 for $t\bar{t}$(+EW) + single top, and 1.0 and 1.6 for $Z/\gamma ^*$+jets backgrounds.

**Table 5:** Numbers of events observed in the signal regions used in the analysis compared with background expectations obtained from the fits described in the text. No signal contribution is considered in the CRs for the fit. Empty cells (indicated by a ‘–’) correspond to estimates lower than 0.01. The p-values ($p_{0}$) give the probabilities of the observations being consistent with the estimated backgrounds. For an observed number of events lower than expected, the p value is truncated at 0.5. Between parentheses, *p*-values are also given as the number of equivalent Gaussian standard deviations (Z). Also shown are 95 % CL upper limits on the visible cross-section ($\langle \epsilon \sigma \rangle _\mathrm{obs}^{95}$), the visible number of signal events ($S_\mathrm{obs}^{95}$ ) and the number of signal events ($S_\mathrm{exp}^{95}$) given the expected number of background events (and $\pm 1\sigma$ excursions of the expectation)

Signal Region	2jl	2jm	2jt	4jt	5j	6jm	6jt
MC expected events							
Diboson	31	31	3.5	0.6	2.1	0.9	0.4
$Z/\gamma ^*$+jets	167	104	13	2.0	5.4	2.8	1.4
*W*+jets	80	46	5.0	1.1	3.4	1.7	1.0
$t\bar{t}$(+EW) + single top	18	17	1.3	0.9	2.7	1.6	1.0
Multi-jet	0.7	0.8	0.04	–	–	–	–
Total MC	296	199	23	4.6	14	7.0	3.8
Fitted background events							
Diboson	$31 \pm 15$	$31 \pm 16$	$3.5 \pm 1.8$	$0.6 \pm 0.3$	$2.1 \pm 1.1$	$0.9 \pm 0.5$	$0.43 \pm 0.27$
$Z/\gamma ^*$+jets	$170 \pm 16$	$114 \pm 11$	$16 \pm 4$	$2.5 \pm 0.9$	$6.0 \pm 1.3$	$3.2 \pm 1.0$	$2.2 \pm 1.0$
*W*+jets	$68 \pm 10$	$35 \pm 9$	$3.5 \pm 1.3$	$0.9 \pm 0.6$	$3.5 \pm 1.3$	$1.9 \pm 0.9$	$1.2 \pm 0.7$
$t\bar{t}$(+EW) + single top	$14 \pm 3$	$10 \pm 3$	$0.7 \pm 0.4$	$0.6 \pm 0.3$	$1.7 \pm 0.9$	$0.9 \pm 0.5$	$0.32 \pm 0.26$
Multi-jet	$0.49 \pm 0.05$	$0.6 \pm 0.4$	$0.02 \pm 0.10$	–	–	–	–
Total bkg	$283 \pm 24$	$191 \pm 21$	$23 \pm 4$	$4.6 \pm 1.1$	$13.2 \pm 2.2$	$6.9 \pm 1.5$	$4.2 \pm 1.2$
Observed	263	191	26	7	7	4	3
$\langle \epsilon \mathrm{\sigma }\rangle _\mathrm{obs}^{95}$ [fb]	16	15	5.2	2.7	1.7	1.7	1.6
$S_\mathrm{obs}^{95}$	44	48	17	8.7	5.4	5.4	5.0
$$S_\mathrm{exp}^{95}$$	$${54}^{+21}_{-14}$$	$$ { 48 }^{ +16 }_{ -10 }$$	$$ { 14.0 }^{ +5.4 }_{ -3.9 }$$	$$ { 6.3 }^{ +2.9 }_{ -1.7 }$$	$$ { 8.7 }^{ +4.2 }_{ -1.9 }$$	$$ { 6.6 }^{ +3.2 }_{ -1.5 }$$	$$ { 5.7 }^{ +2.8 }_{ -1.5 }$$
$p_{0}$ ($\mathrm Z$)	0.50 (0.00)	0.50 (0.00)	0.40 (0.26)	0.17 (0.94)	0.50 (0.00)	0.50 (0.00)	0.50 (0.00)

**Fig. 5:**
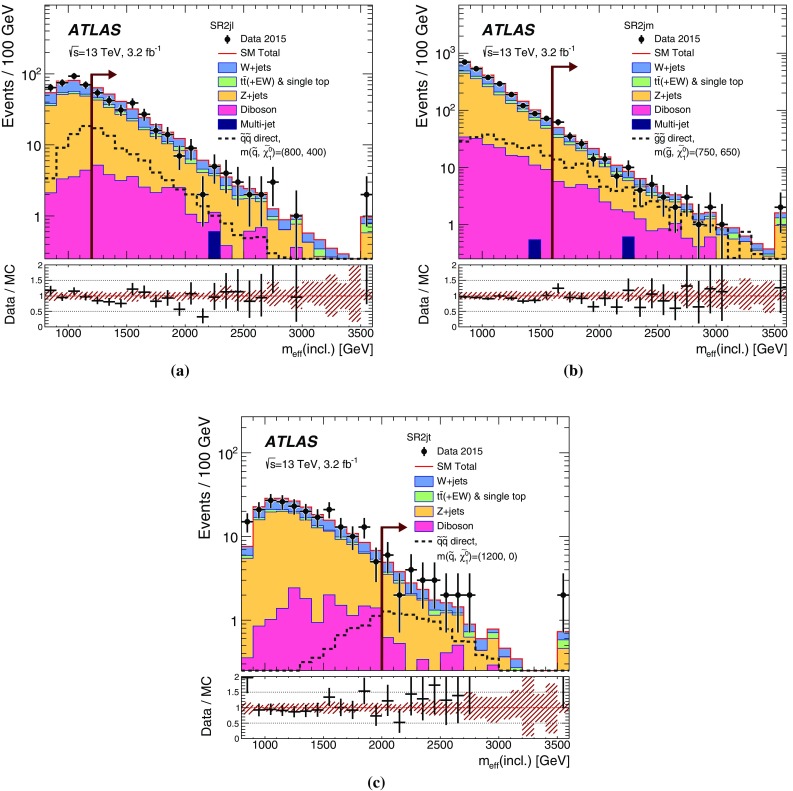
Observed $m_{\mathrm {eff}}(\mathrm{incl.})$ distributions for the **a** 2jl, **b** 2jm, **c** 2jt signal regions. The *histograms* denote the MC background expectations prior to the fits described in the text, normalized to cross-section times integrated luminosity. The last bin includes the overflow. In the *lower panels* the hatched (*red*) error bands denote the combined experimental, MC statistical and theoretical modelling uncertainties. The *arrows* indicate the values at which the requirements on $m_{\mathrm {eff}}(\mathrm{incl.})$ are applied. Expected distributions for benchmark model points, normalized to NLO+NLL cross-section (Sect. 3) times integrated luminosity, are also shown for comparison (masses in ${\mathrm{GeV}}$)

**Fig. 6:**
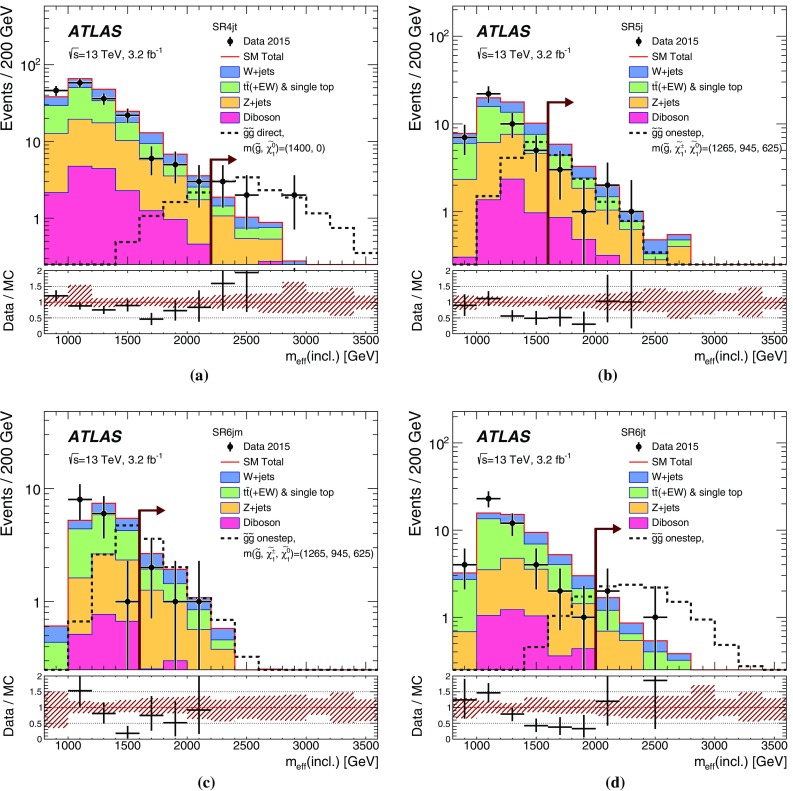
Observed $m_{\mathrm {eff}}(\mathrm{incl.})$ distributions for the **a** 4jt, **b** 5j, **c** 6jm and **d** 6jt signal regions. The histograms denote the MC background expectations prior to the fits described in the text, normalized to cross-section times integrated luminosity. The last bin includes the overflow. In the *lower panels* the hatched (*red*) error bands denote the combined experimental, MC statistical and theoretical modelling uncertainties. The *arrows* indicate the values at which the requirements on $m_{\mathrm {eff}}(\mathrm{incl.})$ are applied. Expected distributions for benchmark model points, normalized to NLO+NLL cross-section (Sect. 3) times integrated luminosity, are also shown for comparison (masses in ${\mathrm{GeV}}$)

Distributions of $m_{\mathrm {eff}}(\mathrm{incl.})$ obtained before the final selections on this quantity (but after applying all other selections), for data and the different MC samples normalized with the theoretical cross-sections, i.e. before applying the normalization from the CR fit, are shown in Figs. 5 and 6. Examples of typical expected SUSY signals are shown for illustration. These signals correspond to the processes to which each SR is primarily sensitive – $\tilde{q} \tilde{q}$ production for the lower jet-multiplicity SRs and $\tilde{g} \tilde{g}$ production for the higher jet-multiplicity SRs. In these figures, data and background distributions largely agree within uncertainties. The differences seen in the lower regions of $m_{\mathrm {eff}}(\mathrm{incl.})$ distribution (1.2 – 2.0 ${\mathrm{TeV}}$) in Fig. 6 do not affect the background expectations in the signal regions since the backgrounds are normalized using control regions (Table 3) with the same $m_{\mathrm {eff}}(\mathrm{incl.})$ selections. The fit to the CRs for each SR compensates for the differences related to the overall normalization of the background seen in Figs. 5 and 6, leading to the good agreements between data and post-fit expectations in the SRs observed in Table 5 and Fig. 4.

In the absence of a statistically significant excess, limits are set on contributions to the SRs from BSM physics. Upper limits at 95 % CL on the number of BSM signal events in each SR and the corresponding visible BSM cross-section are derived from the model-independent fits described in Sect. 5 using the $CL_\mathrm{s}$ prescription. Limits are evaluated using MC pseudo-experiments. The results are presented in Table 5.

The model-dependent fits in all the SRs are then used to set limits on specific classes of SUSY models, using the result from the SR with the best expected sensitivity at each point in each model parameter space. ‘Observed limits’ are calculated from the observed SR event yields for the nominal signal cross-section. ‘Expected limits’ are calculated by setting the nominal event yield in each SR to the corresponding mean expected background.

**Fig. 7:**
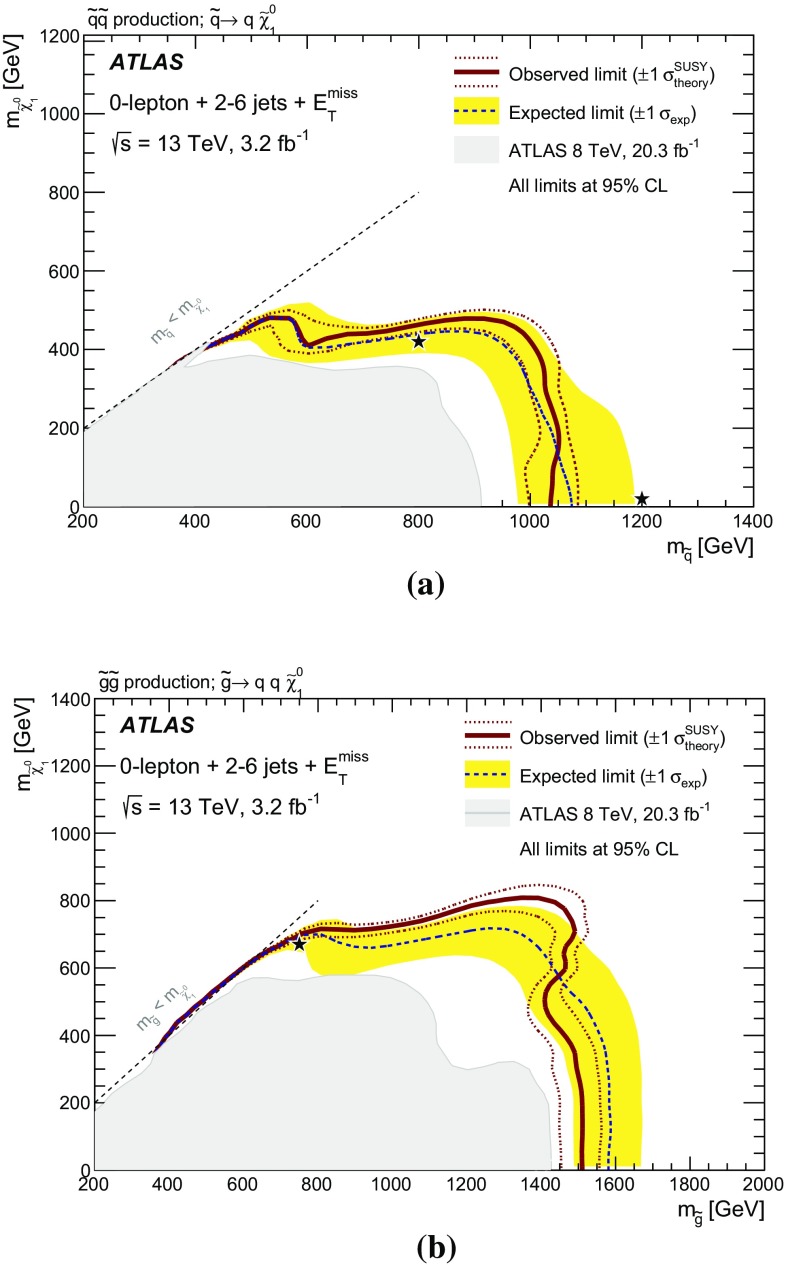
Exclusion limits for direct production of **a** light-flavour squark pairs with decoupled gluinos and **b** gluino pairs with decoupled squarks. Gluinos (light-flavour squarks) are required to decay to two quarks (one quark) and a neutralino LSP. Exclusion limits are obtained by using the signal region with the best expected sensitivity at each point. The *blue dashed lines* show the expected limits at 95 % CL, with the light (*yellow*) bands indicating the $1\sigma$ excursions due to experimental and background-only theoretical uncertainties. Observed limits are indicated by medium dark (*maroon*) curves where the solid contour represents the nominal limit, and the *dotted lines* are obtained by varying the signal cross-section by the renormalization and factorization scale and PDF uncertainties. Results are compared with the observed limits obtained by the previous ATLAS search [15]. The *black stars* indicate the benchmark models used in Figs. 5 and 6

In Fig. 7, limits are shown for two classes of simplified models in which only direct production of light-flavour squark or gluino pairs are considered. In these simplified model scenarios, the upper limit of the excluded light-flavour squark mass region is 1.03 ${\mathrm{TeV}}$ assuming massless $\tilde{\chi }^0_1$, as obtained from the signal region 2jt. The corresponding limit on the gluino mass is 1.51 ${\mathrm{TeV}}$  if the $\tilde{\chi }^0_1$ is massless, as obtained from the signal region 4jt. The best sensitivity in the region of parameter space where the mass difference between the squark (gluino) and the lightest neutralino is small is obtained from the signal region 2jm.

**Fig. 8:**
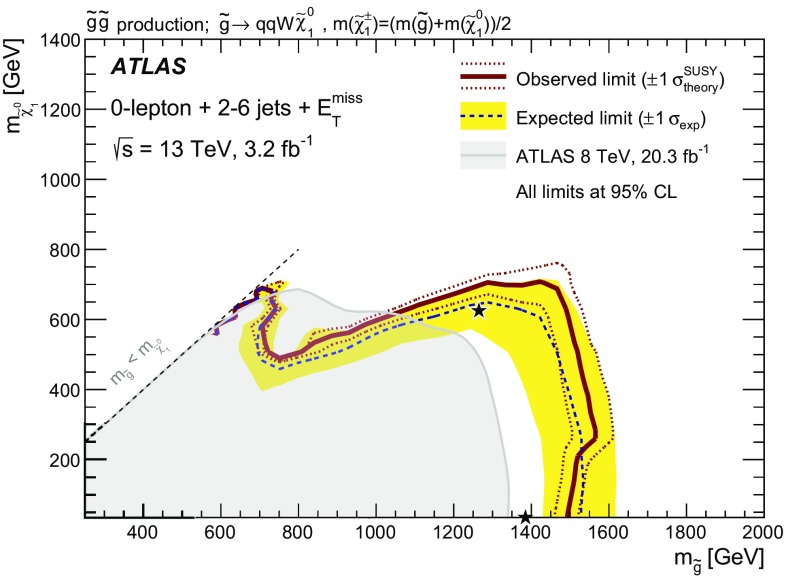
Exclusion limits for pair-produced gluinos each decaying via an intermediate $\tilde{\chi }^\pm _1$ to two quarks, a *W* boson and a $\tilde{\chi }^0_1$ for models with a fixed $m(\tilde{\chi }^\pm _1)=(m(\tilde{g})+m(\tilde{\chi }^0_1))/2$ and varying values of $m(\tilde{g})$ and $m({\tilde{\chi }^0_1})$. Exclusion limits are obtained by using the signal region with the best expected sensitivity at each point. The *blue dashed lines* show the expected limits at 95 % CL, with the light (*yellow*) bands indicating the $1\sigma$ excursions due to experimental and background-only theoretical uncertainties. Observed limits are indicated by medium dark (*maroon*) curves where the solid contour represents the nominal limit, and the *dotted lines* are obtained by varying the signal cross-section by the renormalization and factorization scale and PDF uncertainties. Results are compared with the observed limits obtained from the statistical combination of the search with no lepton and the search with one isolated lepton, high-$p_{\text {T}}$ jets and missing transverse momentum performed at ATLAS [15]. The *black stars* indicate the benchmark models used in Fig. 6

In Fig. 8, limits are shown for pair-produced gluinos each decaying via an intermediate $\tilde{\chi }^\pm _1$ to two quarks, a *W* boson and a $\tilde{\chi }^0_1$. Results are presented for simplified models in which the mass of the chargino $\tilde{\chi }^\pm _1$ is fixed to $m(\tilde{\chi }^\pm _1)=(m(\tilde{g})+m(\tilde{\chi }^0_1))/2$. For a $\tilde{\chi }^0_1$ mass of $\sim$ 200 ${\mathrm{GeV}}$, the lower limit on the gluino mass, obtained from the signal region 4jt, extends up to 1.5 ${\mathrm{TeV}}$ in this model. In the region of parameter space where the mass difference between the gluino and the lightest neutralino is small, the best sensitivity is obtained from the signal region 2jm. Results are compared with the observed limits obtained from the statistical combination of the search with no lepton and the search with one isolated lepton, high-$p_{\text {T}}$ jets and missing transverse momentum performed at ATLAS [15] using the 8 ${\mathrm{TeV}}$ data. Statistical combinations of these two searches, designed to be statistically independent in their signal and control region definitions, are performed in order to increase the exclusion reach in models in which at least two analyses obtain comparable sensitivities, and still provide the strongest exclusion limits in the region of parameter space in which the mass of gluino is between 700 and 1100 ${\mathrm{GeV}}$  and the $\tilde{\chi }^0_1$ mass is above $\sim$ 500 ${\mathrm{GeV}}$.

## Conclusion

This paper reports a search for squarks and gluinos in final states containing high-$p_{\text {T}}$ jets, large missing transverse momentum but no electrons or muons, based on a 3.2 $\mathrm{fb}^{-1}$ dataset of $\sqrt{s}=13 {\mathrm{TeV}}$ proton–proton collisions recorded by the ATLAS experiment at the LHC in 2015. Good agreement is seen between the numbers of events observed in the data and the numbers of events expected from SM processes.

Results are interpreted in terms of simplified models with only light-flavour squarks, or gluinos, together with a neutralino LSP, with the masses of all the other SUSY particles set beyond the reach of the LHC. For a massless lightest neutralino, gluino masses below 1.51 ${\mathrm{TeV}}$ are excluded at the 95 % confidence level in a simplified model with only gluinos and the lightest neutralino. For a simplified model involving the strong production of squarks of the first and second generations, with decays to a massless lightest neutralino, squark masses below 1.03 ${\mathrm{TeV}}$ are excluded, assuming mass-degenerate squarks. In simplified models with pair-produced gluinos, each decaying via an intermediate $\tilde{\chi }^\pm _1$ to two quarks, a *W* boson and a $\tilde{\chi }^0_1$, gluino masses below 1.5 ${\mathrm{TeV}}$ are excluded for $\tilde{\chi }^0_1$ masses of $\sim$ 200 ${\mathrm{GeV}}$. These results substantially extend the region of supersymmetric parameter space excluded by previous LHC searches.

## Footnotes

1 ATLAS uses a right-handed coordinate system with its origin at the nominal interaction point in the centre of the detector. The positive *x*-axis is defined by the direction from the interaction point to the centre of the LHC ring, with the positive *y*-axis pointing upwards, while the beam direction defines the *z*-axis. Cylindrical coordinates $(r,\phi )$ are used in the transverse plane, $\phi$ being the azimuthal angle around the *z*-axis. The pseudorapidity $\eta$ is defined in terms of the polar angle $\theta$ by $\eta =-\ln \tan (\theta /2)$ and the rapidity is defined as $y = (1/2)\ln [(E+p_z)/(E-p_z)]$ where *E* is the energy and $p_\mathrm{z}$ the longitudinal momentum of the object of interest. The transverse momentum $p_{\text {T}}$, the transverse energy $E_{\text {T}}$ and the missing transverse momentum $E_{\text {T}}^{\text {miss}}$ are defined in the *x*–*y* plane unless stated otherwise.

2 Direct decays are those where the considered SUSY particles decay directly into SM particles and the LSP.

3 One-step decays refer to the cases where the decays occur via one intermediate on-shell SUSY particle.
